# ZIF‐67‐Confined Pd Single‐Atom Catalysts Implanted Into Polydopamine‐Modified Bamboo Microchannels for Robust Continuous‐Flow Hydrogenation

**DOI:** 10.1002/advs.202513960

**Published:** 2025-12-07

**Authors:** Sisi Yao, Jiawei Han, Wenjun Zhang, Dengkang Guo, Shenjie Han, Yun Lu, Jingpeng Li

**Affiliations:** ^1^ Engineering Technology Research Center for Building and Decorating Materials of Bamboo State Forestry Administration China National Bamboo Research Center Hangzhou 310012 China; ^2^ Research Institute of Wood Industry Chinese Academy of Forestry Beijing 100091 China; ^3^ Key Laboratory for Advanced Technology in Environmental Protection of Jiangsu Province Yancheng Institute of Technology Yancheng 224051 China; ^4^ School of Chemistry and Chemical Engineering Southeast University Nanjing Jiangsu 211189 China

**Keywords:** bamboo microreactor, catalytic hydrogenation, continuous flow, metal‐organic frameworks, single‐atom catalysts

## Abstract

Developing efficient catalytic hydrogenation microreactors with minimal catalyst loading yet high active‐site density for treating concentrated organic pollutants remains challenging. Herein, a facile room‐temperature method is presented to stabilize Pd single‐atom catalysts (SACs) within ZIF‐67 crystals during their formation, aiming to fabricate an efficient, low‐loading hydrogenation catalytic microreactor (Pd@Z‐P/b CMR). ZIF‐67 is constructed into polydopamine (PDA)‐modified bamboo microchannels under flow conditions, utilizing preimmobilized Co^2+^ seeds in PDA. Despite an ultralow Pd loading (0.0014 wt.%), the Pd@Z‐P/b CMR featuring atomically dispersed Pd─N_4_ sites in ZIF‐67 considerably outperforms its Pd nanoparticle–loaded counterpart (Pd 0.0035 wt.%) in continuous‐flow hydrogenation, demonstrating superior robustness and maximized Pd utilization with 94.3% conversion efficiency for saturated 4‐nitroaniline over 10 d and 97.5% conversion efficiency for gram‐level methylene blue (1.0 g L^−1^) over 5 d. Crucially, it exhibits consistent, high performance in real environmental water samples. The high surface area and porosity of ZIF‐67 stabilize the Pd SACs, enhance dispersion, and ensure accessibility. Simultaneously, the Pd─N_4_ configuration facilitates electron transfer and optimizes hydrogen binding energy, dramatically boosting the catalytic activity. This work provides a novel strategy for room‐temperature SAC synthesis and the effective treatment of high‐concentration organic pollutants for sustainable wastewater remediation.

## Graphical abstract

The report presents a facile room‐temperature method to stabilize Pd single‐atom catalysts within ZIF‐67 crystals during their formation. Despite an ultralow Pd loading (0.0014 wt%), the catalytic microreactor, featuring atomically dispersed Pd‐N_4_ sites in ZIF‐67, demonstrates superior robustness and maximized Pd utilization, significantly outperforming conventional catalysts in the continuous‐flow treatment of high‐concentration organic pollutants.

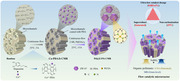

## 1 Introduction

Flow catalytic microreactors (CMRs) enable an efficient, selective, and continuous water remediation by synergistically integrating continuous filtration with heterogeneous catalytic hydrogenation. This confined‐space architecture enhances thermodynamic and kinetic mass transfer within microchannel environments, accelerating diffusion‐reaction processes.^[^ ^1^ ^]^ Among CMRs, those based on natural plants leverage intricate microchannels and efficient fluid transport systems for catalytic applications. In particular, bamboo‐based CMRs have emerged as promising platforms,^[^ ^2^, ^3^, ^4^, ^5^, ^6^ ^]^ with their intrinsic high surface‐area‐to‐volume ratios, superior mass transfer efficiency, abundant active sites, and inherent sustainability offering distinct advantages for continuous‐flow operations.^[^ ^6^, ^7^ ^]^ Nevertheless, CMRs in general suffer from inefficient nanocatalyst utilization owing to poor atomic efficiency^[^ ^8^ ^]^ and limited efficiency for treating high‐concentration pollutants, mainly due to insufficient catalyst stability and performance,^[^ ^9^ ^]^ impeding their full utilization in environmental applications.

Single‐atom catalysts (SACs), i.e., atomically dispersed metal atoms anchored on supports, are promising catalysts that maximize metal atom utilization efficiency.^[^ ^10^, ^11^ ^]^ Unlike conventional metal nanoparticle catalysts, SACs exhibit unique characteristics, such as unsaturated coordination configurations, quantum size effects, and strong atom–support interactions, which result in exceptional catalytic activity, selectivity, and stability.^[^ ^12^ ^]^ Unfortunately, owing to their inherently high surface energy, SACs exhibit high mobility and a strong aggregation tendency, which are detrimental to stability during catalytic reactions. This limitation can be overcome by designing supports that can form robust interactions with isolated metal atoms. With advantages such as abundant coordination sites, structural defects, and tunable porosity, metal–organic frameworks (MOFs) are ideal supports for SACs, enabling effective anchoring of metal atoms via strong interactions or charge transfer, thereby ensuring atomic dispersion and enhanced stability.^[^ ^13^ ^]^ However, the conventional post‐conversion of MOF‐coordinated metal precursors into supported SACs typically requires high‐temperature pyrolysis and reduction, which often degrade the well‐defined porosity of MOFs and adversely affect their composition, morphology, and specific surface area.^[^ ^14^, ^15^ ^]^ Therefore, the development of an alternative and efficient room‐temperature strategy for the immobilization of SACs within MOFs to effectively treat high‐concentration organic pollutants is highly desirable.

Herein, we report a room‐temperature strategy to confine Pd SACs within crystals of the zeolitic imidazolate framework ZIF‐67 during their assembly. By flow‐implanting ZIF‐67 into polydopamine (PDA)‐modified bamboo microchannels using preimmobilized Co^2+^ seeds, the resulting ultralow‐loading (0.0014 wt.%) Pd CMR (Pd@Z‐P/b CMR) outperforms its nanoparticle‐loaded analog (Pd‐Z‐P/b CMR, Pd 0.0035 wt.%) in continuous‐flow hydrogenation, achieving 94.3% conversion efficiency for saturated 4‐nitroaniline (4‐NA) over 10 d and 97.5% conversion efficiency for gram‐level methylene blue (1.0 g L^−1^) over 5 d, even in environmental water samples. This exceptional robustness stems from the porosity of ZIF‐67, which stabilizes the atomically dispersed Pd─N_4_ sites, maximizing atomic dispersion, accessibility, and electron transfer while optimizing hydrogen binding for enhanced activity. Despite the extremely low Pd loading, the bamboo‐based CMR demonstrates remarkable efficiency in continuously treating high‐concentration organic pollutants even at saturation levels, representing a groundbreaking advancement in plant‐based CMRs.

## 2 Results and Discussion

### 2.1 Synthesis and Structural Characterization of the Pd@Z‐P/b CMR

**Figure 1:**
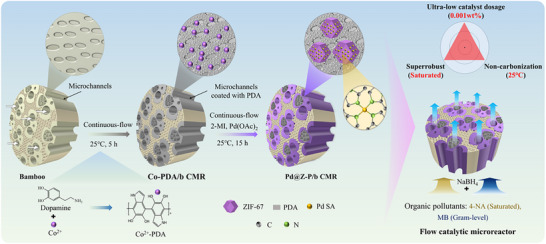
Schematic of the preparation progress of the Pd@Z‐P/b CMR for the continuous‐flow catalytic hydrogenation of high‐concentration organic pollutants.

Living bamboo transports water and inorganic salts upward from the ground through its xylem microchannels via transpiration, as well as nutrients over long distances through its phloem microchannels via capillary action. The long microchannels in the bamboo vascular tissue exhibit a high surface‐area‐to‐volume ratio exceeding 30000 m^2^ m^−3^, which perfectly aligns with the design requirements (10000–50000 m^2^ m^−3^) for flow CMRs, making bamboo one of the most promising candidates for their fabrication. Drawing inspiration from the internal fluid transport mechanism of bamboo, ZIF‐67‐confined Pd SACs were integrated into PDA‐modified microchannels within bamboo to develop a bamboo‐based CMR (Pd@Z‐P/b CMR) for the continuous‐flow catalytic hydrogenation of high‐concentration organic pollutants (**Figure** **1**). Dopamine molecules were immobilized on the microchannel walls of bamboo using a flow self‐assembly technique, and Co^2+^ ions were concurrently integrated into the hierarchical structures of PDA. As a result, the transport microchannels of bamboo changed from their original color to a dark brown. This process decreases the reaction time compared with the previously reported two‐step method.^[^ ^16^, ^17^, ^18^ ^]^ Subsequently, Pd SACs were synchronously incorporated into ZIF‐67 crystals during the formation and anchoring of ZIF‐67 onto the microchannel walls of the bamboo vascular bundles, resulting in a color transition to modena, thereby enabling the successful fabrication of a bamboo‐based single‐atom CMR. Notably, this preparation strategy can be achieved under ambient conditions, without complex experimental setups or high‐intensity processes such as vacuum treatment,^[^ ^19^ ^]^ carbonization,^[^ ^20^, ^21^ ^]^ or hydrothermal reactions,^[^ ^22^ ^]^ reducing the fabrication cost of the CMR.

**Figure 2:**
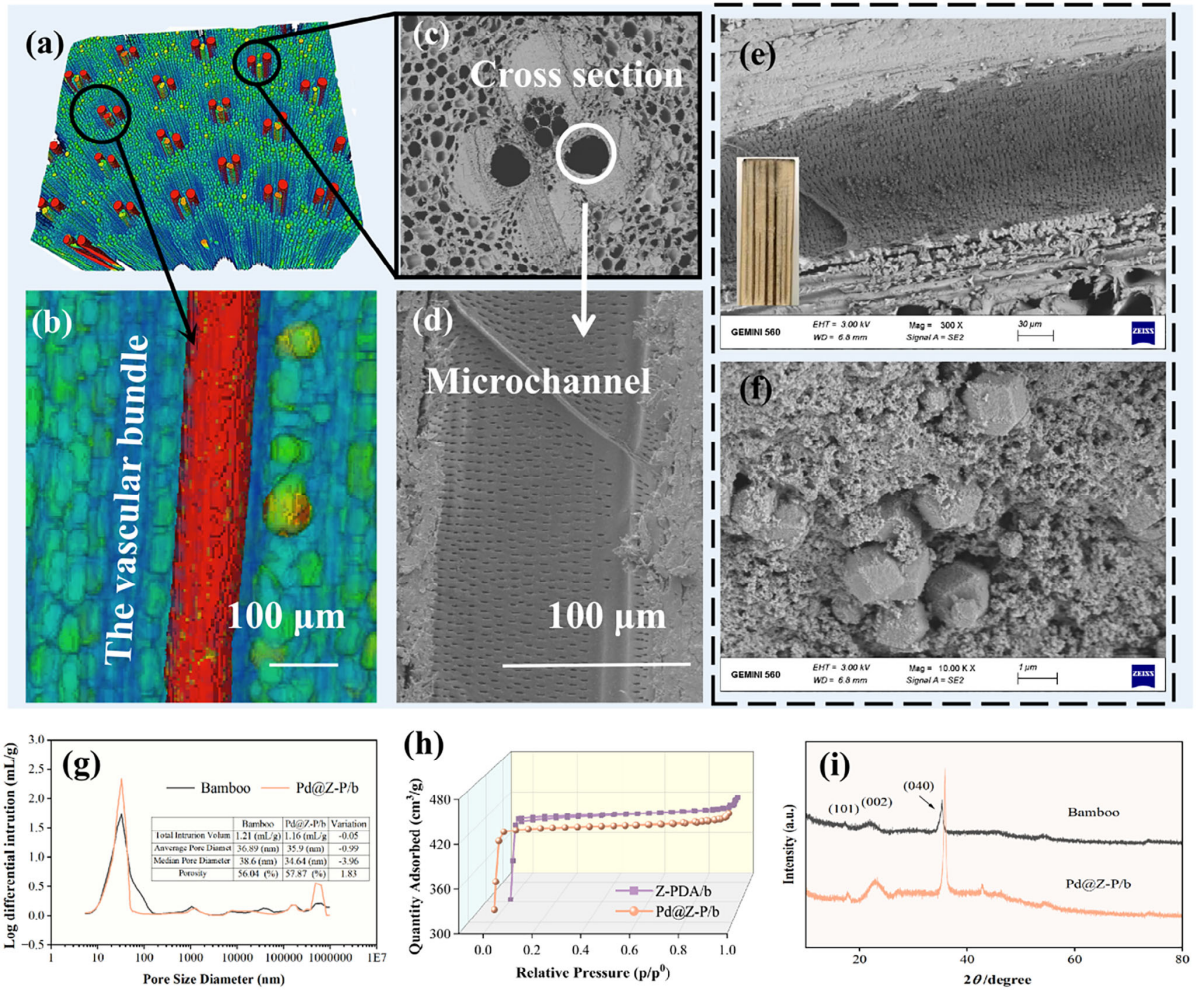
Microcomputed tomography images of a) the cross‐section and b) Vascular bundle in the longitudinal section of bamboo. SEM images of the bamboo vascular bundle: c) cross‐section and d) longitudinal section. e,f) SEM images of a microchannel in the Pd@Z‐P/b CMR at various magnifications. The inset in Figure 2e shows the longitudinal cross‐section of the physical structure of the Pd@Z‐P/b CMR. g) Mercury intrusion porosimetry analysis of original bamboo and the Pd@Z‐P/b CMR. h) N_2_ sorption isotherms of the Z‐PDA/b and Pd@Z‐P/b CMRs. i) XRD patterns of original bamboo and the Pd@Z‐P/b CMR.

Bamboo strips were pretreated using a bamboo wire drawing machine to generate uniform cylindrical bamboo materials with no apparent surface defects, as depicted in Figure S1 (Supporting Information). **Figure** **2**a,b presents 3D microcomputed tomography images and a partially enlarged illustration of the bamboo specimen, which clearly shows that the microchannels are entirely hollow along their entire length. The bamboo cross‐section image shown in Figure 2c reveals a fully intact vascular bundle system comprising two metaxylem vessels, fibers, and metaphloem sieve tubes along with their associated companion cells.^[^ ^23^, ^24^ ^]^ The average diameter of the metaxylem vessels, which function as the primary transport microchannels, is 126 µm. Figure 2d,e shows scanning electron microscopy (SEM) images of a bamboo microchannel before and after deposition of Pd SACs. The inset in Figure 2e presents the color change only at the microchannel positions, suggesting that the catalyst is predominantly localized within the microchannels. Figure 2f shows that the ZIF‐67 crystals, featuring a regular dodecahedral structure, are uniformly dispersed within the PDA‐modified bamboo microchannels. Moreover, the PDA compound structures wrapped around the ZIF‐67 crystals effectively adhere and disperse them, thereby reducing catalyst loss during the continuous‐flow catalytic process.

To further investigate the microchannel structure characteristics of the Pd@Z‐P/b CMR, a comprehensive analysis was performed using mercury intrusion porosimetry (Figure 2g; Figure S2, Supporting Information) and BET measurements (Figure 2h). Compared with the original bamboo, the Pd@Z‐P/b CMR exhibits a slight increase in porosity, particularly in pores of ≈50 nm, and a 2.68% reduction in average pore size. This is attributable to the formation of a novel pore structure upon loading ZIF‐67 and PDA onto the bamboo microchannel walls.^[^ ^25^, ^26^ ^]^ N_2_ sorption isotherms (Figure 2h) further demonstrate that the porous structure of ZIF‐67 crystals is effectively retained after introducing Pd SACs, aligning with previous research.^[^ ^27^ ^]^ The X‐ray diffraction (XRD) patterns of untreated bamboo and the Pd@Z‐P/b CMR (Figure 2i) exhibit comparable crystalline diffraction peaks at ≈16°, 22°, and 35°, corresponding to the (101), (002), and (040) planes.^[^ ^28^ ^]^ No distinct ZIF‐67 or Pd peaks are observed, owing to their low concentrations (Pd: 0.0014 wt.%; Co: 0.0663 wt.%, Table S1, Supporting Information) within the bamboo microchannels. However, the distinct ZIF‐67 crystal peaks shown in Figure S3 (Supporting Information) confirm the successful synthesis. The absence of Pd peaks indicates that the embedded Pd atoms are single atoms and not crystalline aggregates, thereby preserving the crystallinity and structure of ZIF‐67.^[^ ^29^ ^]^

### 2.2 Atomic‐Scale Characterization of Pd SACs

**Figure 3:**
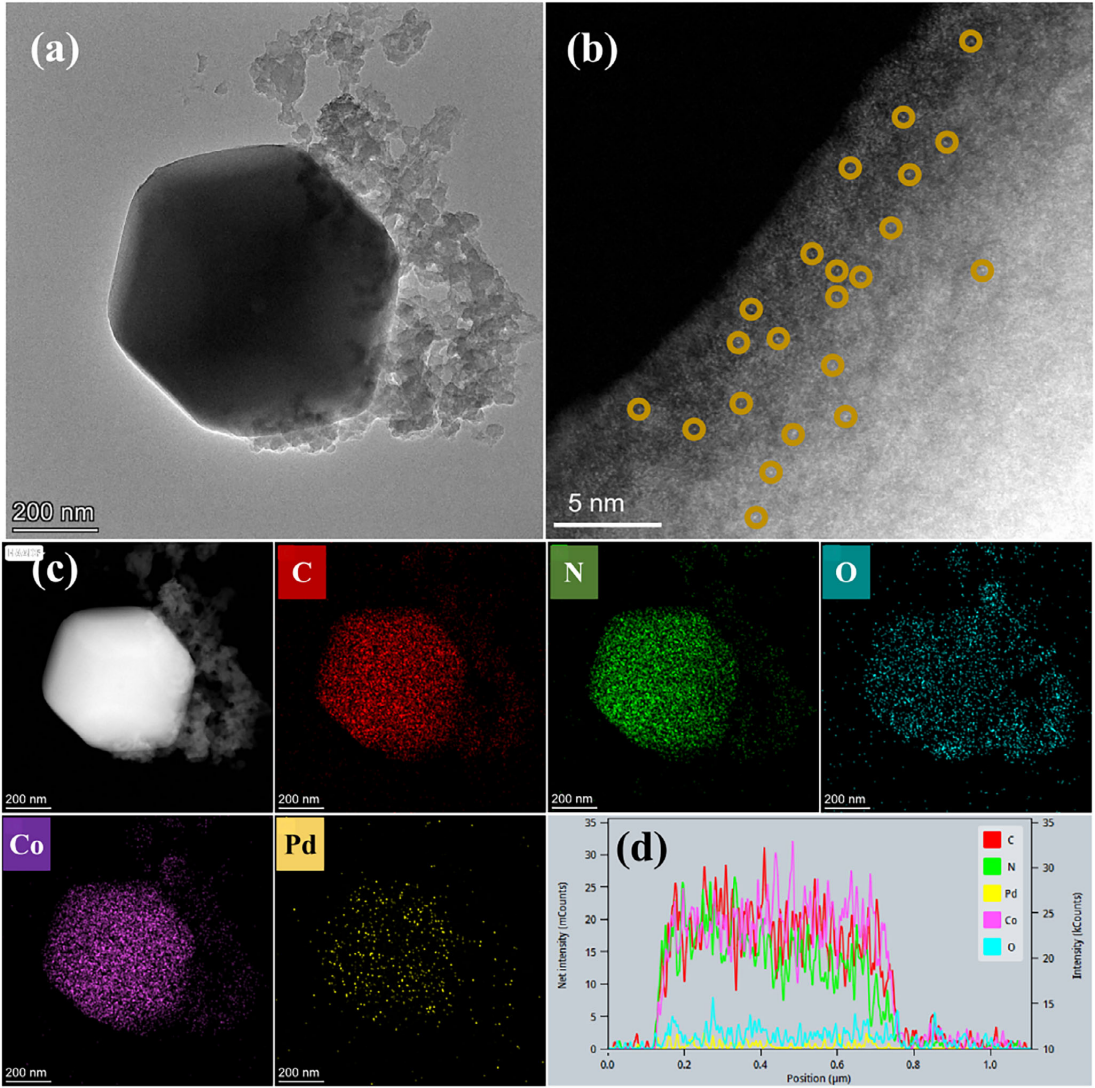
a) TEM, b) AC‐HAADF‐STEM, c) EDS area scan, and d) line scan images of the Pd@Z‐P catalyst.

To determine the oxidation state of metallic Pd in the Pd@Z‐P/b CMR, transmission electron microscopy (TEM) was used to examine the morphology and particle size. As shown in **Figure** **3**a and Figure S4a (Supporting Information), the catalyst consists of regular dodecahedral ZIF‐67 crystals encapsulated by abundant PDA particles, and their diffraction rings exhibit an amorphous nature (Figure S4b, Supporting Information, inset). However, the TEM image does not show metal nanoparticles, likely owing to resolution limitations. The presence of abundant Pd SACs was confirmed via aberration‐corrected high‐angle annular dark‐field scanning TEM (AC‐HAADF‐STEM), which is a highly effective technique for identifying individual heavy atoms within a localized region. In Figure 3b, numerous uniformly distributed small bright spots (encircled in yellow) reveal atomically dispersed Pd within the ZIF‐67 framework. This was confirmed by energy‐dispersive X‐ray spectroscopy (EDS) mapping (Figure 3c), which shows a homogeneous distribution of Pd, Co, C, N, and O elements, with Pd and Co localized specifically on ZIF‐67 and not in the PDA matrix. For comparison, Pd‐Z‐P catalysts prepared by introducing the Pd(OAc)_2_ precursor solution in the final step were analyzed by TEM and EDS. Figure S5 (Supporting Information) shows metallic Pd aggregated into nanoparticles and predominantly residing on the surface of ZIF‐67. The lattice fringes with a spacing of 0.22 nm correspond to the (111) plane of the face‐centered cubic Pd(0) structure. This confirms that the addition time of the Pd(OAc)_2_ precursor plays a critical role in the formation of Pd atoms and their spatial distribution. The EDS mapping in Figure S6 (Supporting Information) shows that the C, Co, and N elements that constitute the MOF were predominantly distributed within the ZIF‐67 framework. In contrast, Pd nanoparticles were located within the ZIF‐67 framework and on the surface of the PDA matrix. This indicates a stronger interaction between ZIF‐67 and Pd SACs than between ZIF‐67 and Pd.^[^ ^30^ ^]^

**Figure 4:**
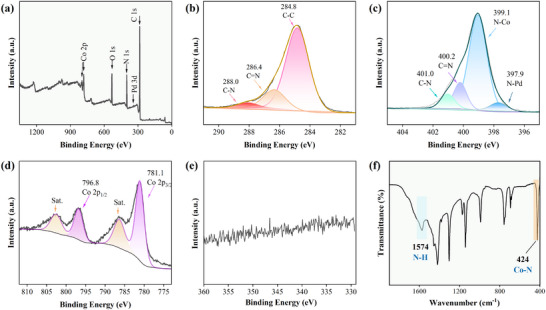
a) Survey XPS spectrum, high‐resolution b) C 1s, c) N 1s, d) Co 2p, and e) Pd 3d XPS spectra, and f) FTIR spectrum of the Pd@Z‐P catalyst.

The surface chemical states of the Pd single‐atom‐based catalysts were systematically analyzed using X‐ray photoelectron spectroscopy (XPS) and FTIR (**Figure** **4**). The survey XPS spectrum shows peaks at 284.9, 399.1, and 531.7 eV (Figure 4a), ascribable to C 1s, N 1s, and O 1s, respectively. The C 1s spectrum was deconvoluted into three components: C─C (284.8 eV), C═N (286.4 eV), and C─N (288.0 eV) bonds originating from ZIF‐67 (Figure 4b).^[^ ^31^ ^]^ Similarly, the N 1s spectrum primarily consists of four components derived from ZIF‐67: C─N (401.0 eV), C═N (400.2 eV), N─Co (399.1 eV), and N─Pd (397.9 eV) bonds (Figure 4c). The N─Pd bond confirms the atomic interaction between ZIF‐67 linkers and Pd SACs.^[^ ^32^, ^33^, ^34^ ^]^ As depicted in Figure 4d, the binding energies of Co(II) 2p_3/2_ and Co(II) 2p_1/2_ at 781.1 and 796.8 eV, respectively, confirm the presence of Co(II). The corresponding satellite peaks are observed at 786.3 and 802.6 eV.^[^ ^25^, ^35^ ^]^ The FTIR analysis further confirmed the chemical state of the Co element (Figure 4f): peaks at 900–1350 and 500–800 cm^−1^ correspond to the in‐plane and out‐of‐plane vibrations of the imidazole ring, respectively, and those at 1350–1500 and 1574 cm^−1^ are assigned to the ring stretching and N─H stretching modes. The peak at 472 cm^−1^ corresponds to Co─N stretching, confirming that Co primarily resides in ZIF‐67.^[^ ^36^ ^]^ The Pd 3d XPS spectrum shows no Pd signal, which could be attributed to the low content of Pd and its presence in the form of single atoms (Figure 4e). This result agrees with previous findings.^[^ ^37^, ^38^ ^]^ Conversely, the XPS spectrum of Pd‐Z‐P shows three Pd peaks at 344.2 eV (Pd 3d_5/2_), 339.0 eV (Pd 3d_3/2_), and 335.0 eV (Pd^0^) (Figure S7, Supporting Information).^[^ ^39^, ^40^, ^41^ ^]^

**Figure 5:**
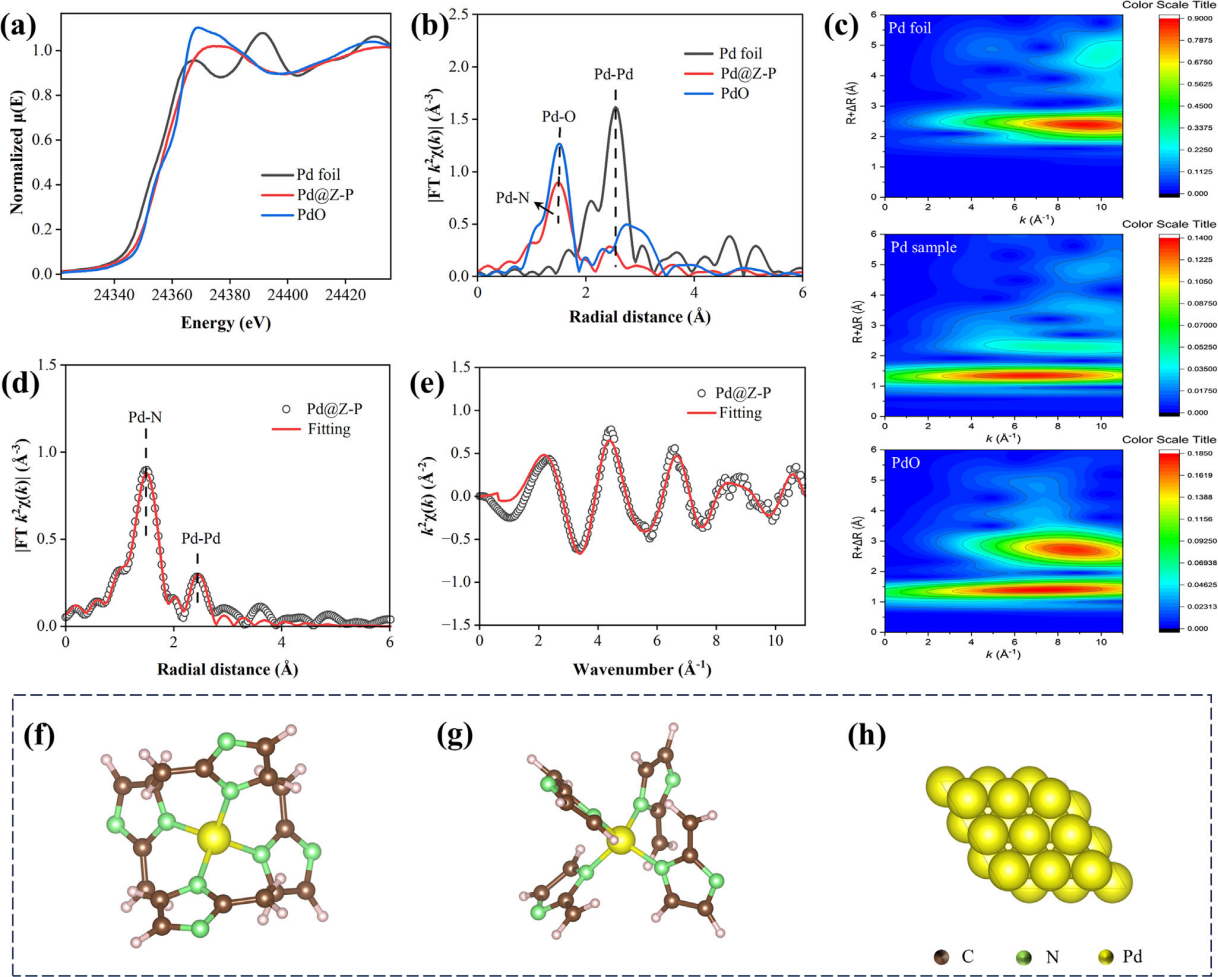
a) XANES spectra, b) EXAFS spectra, and c) wavelet transform analysis of the Pd@Z‐P catalyst, Pd foil, and PdO, respectively. EXAFS fitting curves of the Pd@Z‐P catalyst in d) K space and e) R space. Geometric configurations of f,g) the Pd@Z‐P catalyst and h) Pd (111).

The coordination structures of Pd species in the Pd@Z‐P catalyst were further analyzed using synchrotron‐radiation‐based X‐ray absorption fine structure (XAFS) spectroscopy. The Pd K‐edge X‐ray absorption near‐edge structure (XANES) spectra (**Figure** **5**a) indicate that the valence state of the Pd absorption edge in the Pd@Z‐P catalyst lies between those of Pd foil and PdO, being closer to that of PdO. This confirms the +*δ* charge state of Pd.^[^ ^42^ ^]^ The EXAFS spectra exhibit a single peak at 1.5 Å, corresponding to the first Pd─N coordination shell layer (Figure 5b). A minor peak observed at ≈2.5 Å suggests the presence of Pd─Pd interactions. These results demonstrate that Pd predominantly exists as dispersed single atoms in the Pd@Z‐P catalyst, as confirmed by the wavelet transformation analysis of the Pd K‐edge EXAFS signals.^[^ ^43^ ^]^ Thus, Figure 5c shows a distinct hot spot at a K value of ≈6.6 Å^−1^ and an R value of 1.4 Å. This position differed from that of the Pd─Pd scattering hotspot in Pd foil (K = 9.3 Å^−1^ and R = 2.4 Å). Meanwhile, PdO exhibited two hotspots at approximately K = 7.3 Å^−1^, R = 1.4 Å, and K = 8.6 Å^−1^, R = 2.7 Å, corresponding to the first and second shells of the Pd─O scattering signals, respectively. To further investigate the structural parameters of Pd sites in the Pd@Z‐P catalyst, EXAFS curve fitting was performed (Figure 5d,e). According to the results (Table S2, Supporting Information), the coordination number of Pd with N was 3.8, dominating the first shell of the Pd@Z‐P catalyst. This result suggests that the Pd─N_4_ configuration is the predominant species in the Pd@Z‐P catalyst (Figure 5f–h).^[^ ^15^, ^44^ ^]^

### 2.3 Continuous‐Flow Catalytic Performance

The catalytic activity of the Pd@Z‐P/b CMR under continuous‐flow conditions was evaluated using the reduction of saturated 4‐NA to 4‐aminophenol (4‐AP) as a model reaction, with NaBH_4_ as the reducing agent. The catalytic conversion of the reactants was analyzed using UV–vis spectroscopy. The results indicate that neither the original bamboo nor the PDA matrix possesses catalytic activity (Figure S8, Supporting Information).

**Figure 6:**
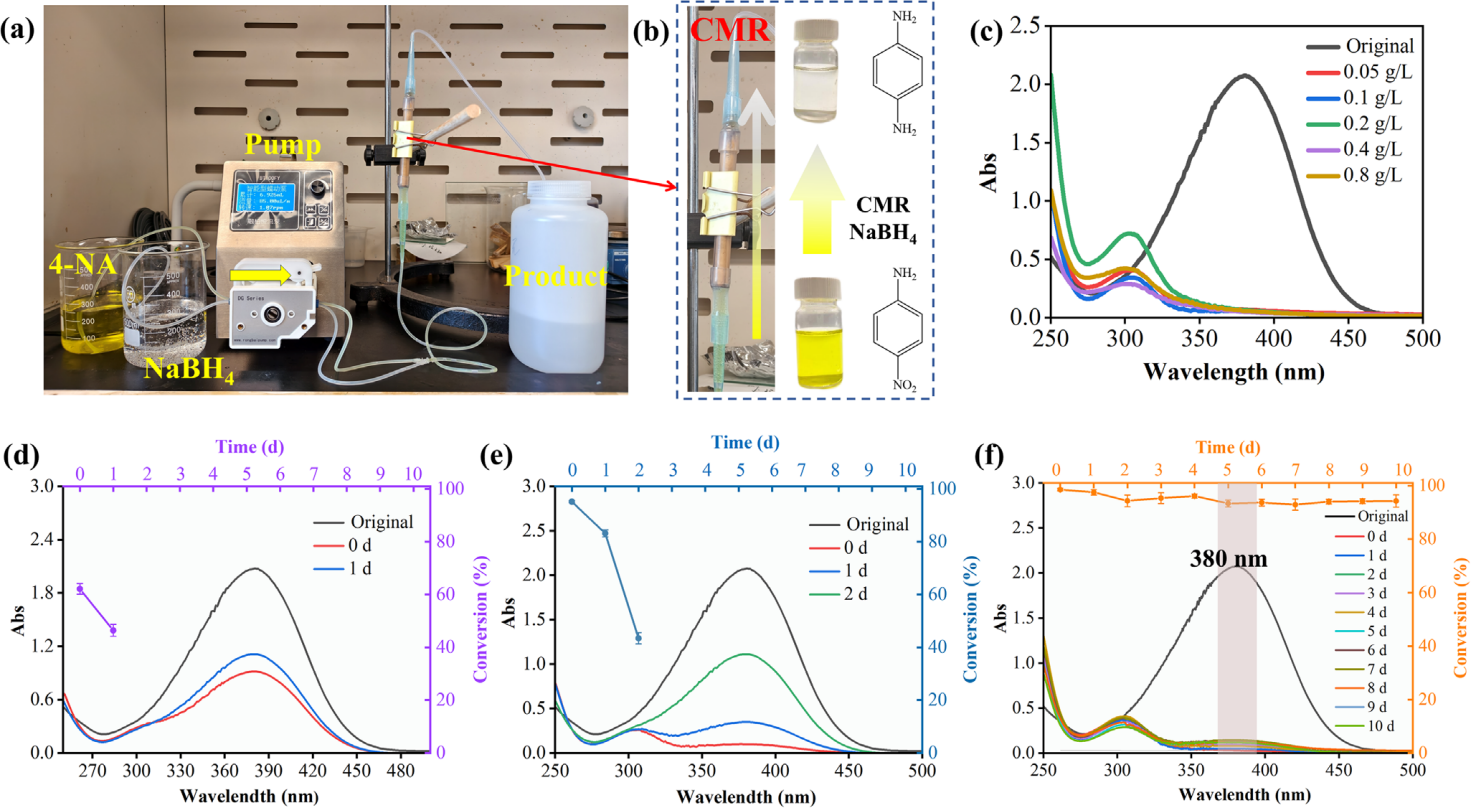
Catalytic performance evaluations under continuous‐flow conditions. a) Home‐made catalytic device. b) Catalytic reduction process of 4‐NA using the CMR. c) Catalytic hydrogenation of 4‐NA at various concentrations. Catalytic performance of the d) Z‐PDA/b, e) Pd‐Z‐P/b, and f) Pd@Z‐P/b CMRs in the reduction of saturated 4‐NA. Data are presented as mean ± SD. n = 5 per group. A maximum single deviation of the data from the mean within 3% is considered acceptable.

**Figure** **6**a shows a specially designed experimental apparatus for continuous‐flow catalytic reduction. In this setup, two solutions of predetermined concentrations are simultaneously pumped through identical silicone tubes at a constant flow rate. The solutions meet at the opposite end of the pump via a three‐way connector and then converge into a tube of larger diameter. This design prevents backflow during mixing while enabling long‐distance transport to the Pd@Z‐P/b CMR. Critically, avoiding conventional premixing by using the three‐way connector minimizes premature reactant contact, thereby eliminating concentration gradients and enhancing accuracy. To ensure unidirectional liquid flow, the side of the Pd@Z‐P/b CMR is sealed using a heat‐shrink tube. As depicted in Figure 6b, the inlet of the Pd@Z‐P/b CMR appeared yellow because of the 4‐NA/NaBH_4_ mixture and turned colorless at the outflow after ≈15–16 s, indicating the occurrence of catalysis.

Initially, a low 4‐NA concentration (0.05 g L^−1^) was employed to investigate the flow catalytic performance of the Pd@Z‐P/b CMR. The flow rate of the reaction solution was accurately controlled at 0.17 mL min^−1^ using a peristaltic pump. The UV–vis absorption spectrum shown in Figure 6c exhibits a substantial intensity reduction for the signal at 380 nm and a new absorption peak at 300 nm. These observations suggest that the 4‐NA concentration was effectively reduced within the Pd@Z‐P/b CMR. Complete catalytic conversion was still achieved at a 4‐NA concentration of 0.1 g L^−1^ (Figure 6c). It is worth noting that only a limited number of reported CMRs can process such a high 4‐NA concentration.^[^ ^9^, ^45^ ^]^ More remarkably, the Pd@Z‐P/b CMR exhibited efficient catalytic activity in 4‐NA reduction even at saturated concentrations (0.8 g L^−1^). To date, no report has shown that CMRs can treat a saturated 4‐NA solution under continuous‐flow conditions. The 4‐NA concentration of 0.8 g L^−1^ was selected for subsequent experiments.

Next, the catalytic Pd@Z‐P/b CMR components that contribute to its efficient catalytic performance were investigated. Under identical continuous operation conditions, the catalytic performance in the reduction of saturated 4‐NA was evaluated using the Z‐PDA/b and Pd‐Z‐P/b CMRs. The UV–vis spectrum (Figure 6d) reveals that the peak at 380 nm was not completely attenuated when the saturated 4‐NA solution flowed through the Z‐PDA/b CMR. Conversely, the peak at 380 nm was entirely attenuated for the Pd‐Z‐P/b CMR (Figure 6e), which indicates that Pd nanoparticles play a crucial role in the catalytic activity of Pd‐Z‐P/b CMR. However, the conversion efficiency of the Pd‐Z‐P/b CMR decreased to 83% after 1 d of continuous operation and further to 43.3% after 2 d. These results suggest that the Pd‐Z‐P/b CMR fails to exhibit sustained long‐term catalytic performance. In contrast, for the Pd@Z‐P/b CMR, after continuous operation for 10 d, only the absorption peak of 4‐phenylenediamine (4‐PD) was observed at ≈300 nm (Figure 6f). The conversion efficiency of saturated 4‐NA remained consistently above 94.3%. Despite its ultralow Pd loading of 0.0014 wt.% (Table S1, Supporting Information), the SAC‐based Pd@Z‐P/b CMR exhibits superior performance and robustness in continuous‐flow catalytic hydrogenation to the nanosized Pd‐loaded Pd‐Z‐P/b CMR with a Pd content of 0.0035 wt.%. This performance enhancement stems from maximized Pd utilization in the Pd@Z‐P/b CMR, confirming the dominant role of Pd SACs in catalytic hydrogenation. Mechanistically, Pd SACs mediate the cleavage of B─H bonds in NaBH_4_, forming Pd─H intermediates with H radicals (H^•^) that attack nitroaromatics at the N^+^ sites, enabling an efficient hydrogenation.

After the catalytic experiment, the recovered Pd@Z‐P/b CMR exhibited dry shrinkage, with up to a 7.02% reduction in diameter after 10 d (Figure S9, Supporting Information). To gain insight into the structural changes of the Pd@Z‐P/b CMR, SEM and TEM analyses were performed. As shown in Figure S10 (Supporting Information), the microstructure of the bamboo‐based material after 10 d of reaction remained stable, with only the parenchyma cells showing minor structural collapse (Figure S10c,d, Supporting Information). Conversely, the Pd@Z‐P catalyst underwent a noticeable morphological change, characterized by a blurring of its peripheral contours. This can be attributed to surface erosion caused by the flushing of the reaction solution under continuous‐flow conditions. However, no metal nanoparticles were observed in the TEM images of the used Pd@Z‐P catalyst (Figures S11 and S12, Supporting Information), and its diffraction rings revealed an amorphous nature. In addition, the concentration of Pd species in the filtrate was monitored during the entire process using inductively coupled plasma mass spectrometry (ICP‐MS) to assess the leaching of Pd from the Pd@Z‐P/b CMR (Figure S13, Supporting Information). The results revealed that the Pd@Z‐P/b CMR exhibited enhanced stability with an initial Pd concentration in the filtrate of only 1.9 ppb, lower than that observed for the conventional Pd/bamboo‐based CMR in our previous study and close to the maximum contaminant limit (5 µg L^−1^) set for drinking water quality standards.^[^ ^7^, ^45^ ^]^

To further confirm the chemical stability of the Pd@Z‐P/b CMR, XRD and XPS measurements of the post‐reaction CMR were conducted (Figures S14–S17, Supporting Information). After 10 days of continuous operation, the characteristic cellulose peak shifted to the left and its height decreased (Figure S14, Supporting Information). The oxygen‐to‐carbon (O/C) ratios were determined to be 0.44 before the reaction and 0.43 after 10 d of reaction. After the reaction, the positions of the characteristic peaks in the high‐resolution XPS spectra of C 1s and O 1s shifted slightly. These results indicate that during long‐term continuous‐flow catalysis, some components of the bamboo matrix, such as lignin and hemicellulose, may be lost into the reaction liquid.^[^ ^46^, ^47^, ^48^ ^]^

### 2.4 Catalytic Activity of Different Coordination Environments

**Figure 7:**
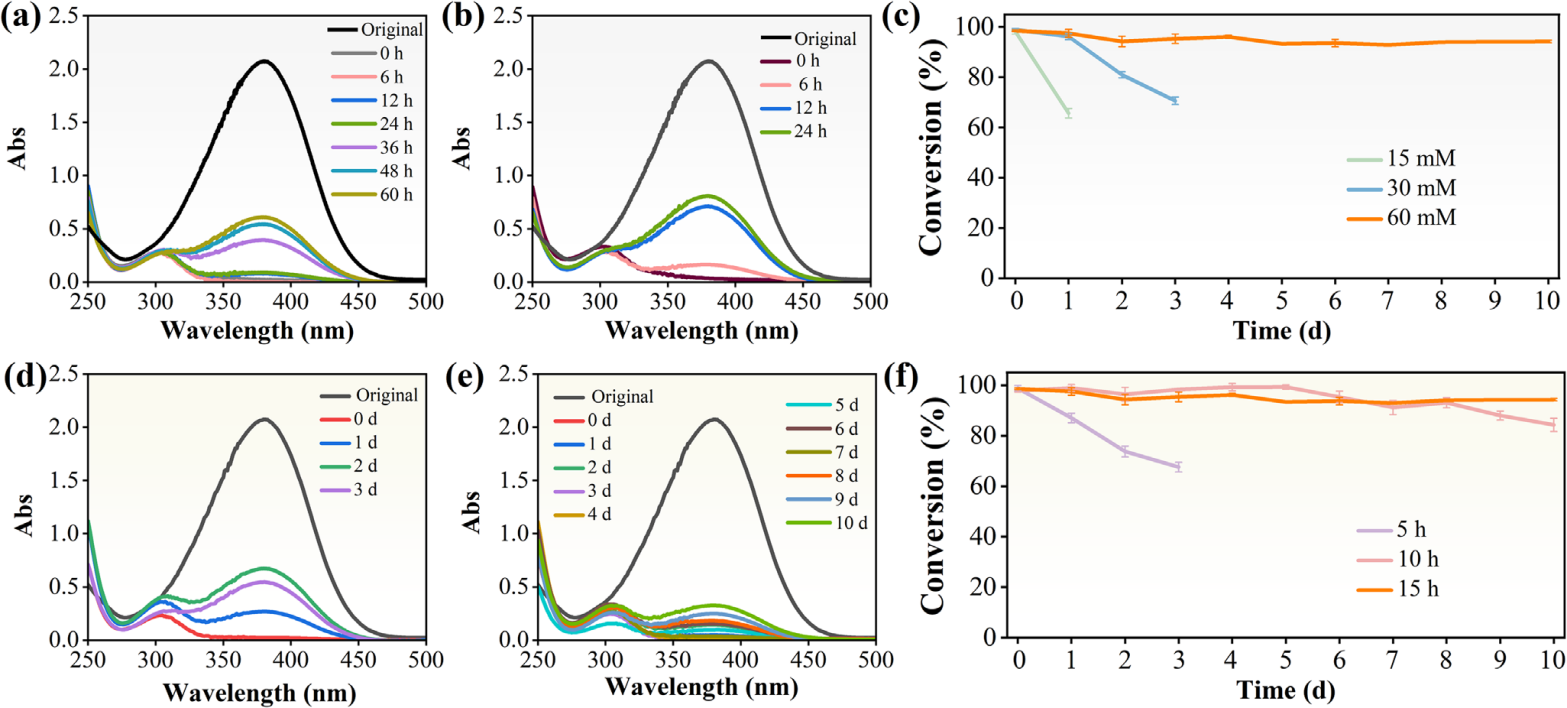
Catalytic performance of Pd@Z‐P/b CMRs prepared with a) 10 mm and b) 30 mm Co(NO_3_)_2_·6H_2_O, and c) the corresponding conversion efficiencies. Catalytic performance of Pd@Z‐P/b CMRs prepared at loading times of Pd precursors of d) 5 h, e) 10 h, and f) the corresponding conversion efficiencies. Data are presented as mean ± SD. n = 5 per group. A maximum single deviation of the data from the mean within 3% is considered acceptable.

To investigate the catalytic activity of the single‐atom sites with varying coordination environments in saturated 4‐NA reduction, Pd@Z(10)‐P/b and Pd@Z(30)‐P/b CMRs were prepared using different concentrations of Co(NO_3_)_2_·6H_2_O. In addition, Pd(5)@Z‐P/b and Pd(10)@Z‐P/b CMRs were synthesized with varying immersion durations in the Pd precursor solution. As shown in **Figure** **7**a–c, during the initial stage of the experiment, the 4‐NA absorption peak at 380 nm in the UV–vis spectrum decreased considerably from its initial value. At this stage, the 4‐NA conversion rate reached nearly 99% for the Pd@Z(10)‐P/b and Pd@Z(30)‐P/b CMRs. However, after 12 h of continuous operation, the 4‐NA conversion rate dropped markedly to 65.7% for the Pd@Z(10)‐P/b CMR. Furthermore, the conversion rate declined rapidly after 36 h of continuous operation for the Pd@Z(30)‐P/b CMR, eventually stabilizing at 70.9% on the third day. This behavior can be attributed to the effect of the 2‐methylimidazole (2‐MI)‐to‐Co source ratio on the crystalline structure of ZIF‐67 (Figure S18, Supporting Information).^[^ ^46^ ^]^ Therefore, a ZIF‐67 catalyst with an appropriate crystalline structure is essential for achieving an efficient, highly robust catalytic process with the Pd@Z‐P/b CMR.

For the Pd(5)@Z‐P/b and Pd(10)@Z‐P/b CMRs, the 4‐NA conversion rate was close to 99% at the beginning of the experiment (Figure 7d–f). After 1 d of continuous operation, the 4‐NA conversion rate over the Pd(5)@Z‐P/b CMR began to decline sharply, decreasing to 73.2% on the second day. In contrast, the catalytic efficiency of the Pd(10)@Z‐P/b CMR for 4‐NA remained above 90% over 6 d of operation. However, from that point onward, the conversion rate gradually decreased, reaching 84.3% on the tenth day. These results highlight the critical importance of appropriately regulating the Pd content in the Pd@Z‐P/b CMR to ensure an efficient and sustained 4‐NA reduction.

### 2.5 Catalyst Stability Toward Various Organic Pollutants

To investigate the universality of the Pd@Z‐P/b CMR, the catalytic performance was systematically evaluated using various nitroaromatic compounds, i.e., 2‐nitroaniline (2‐NA) and 4‐nitrophenol (4‐NP), and the methyl orange (MO) and methylene blue (MB) azo dyes at gram‐level concentrations as representative organic pollutants. Specifically, solutions containing 2‐NA (1.1 g L^−1^, saturated concentration at 25 °C), 4‐NP (0.5 g L^−1^), MB (1.0 g L^−1^), and MO (2.5 g L^−1^) were individually mixed with NaBH_4_ (0.375 M) in equal volume ratios using a three‐way connector. The resulting mixtures were passed through the Pd@Z‐P/b CMR at a flow rate of 0.17 mL min^−1^. The changes in their characteristic absorption peaks were spectrophotometrically monitored at 412 (2‐NA), 400 (4‐NP), 462 (MO), and 664 nm (MB), respectively.

**Figure 8:**
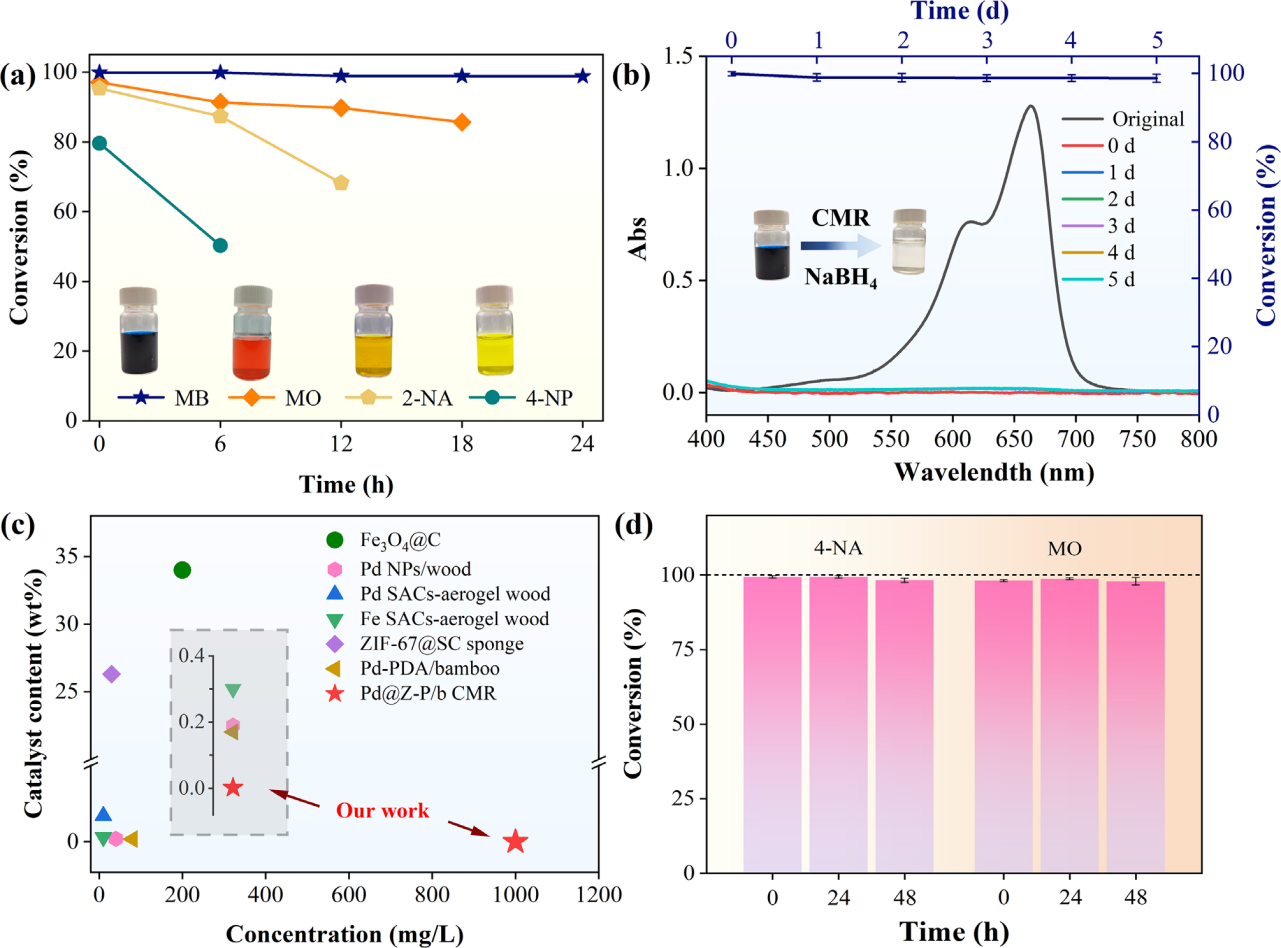
a) Catalytic activity of the Pd@Z‐P/b CMR toward MB, MO, 2‐NA, and 4‐NP. b) Catalytic durability of the Pd@Z‐P/b CMR toward MB. c) Comparative assessment of the catalytic efficiency of bamboo‐based microreactors relative to other previously reported microreactors: Fe_3_O_4_@C,^[^ ^48^ ^]^ Pd NP/wood,^[^ ^49^ ^]^ Pd SACs‐aerogel wood, Fe SACs‐aerogel wood,^[^ ^18^ ^]^ ZIF‐67@SC sponge,^[^ ^50^ ^]^ Pd‐PDA/bamboo.^[^ ^7^ ^]^ d) Renewable performance assessment of the Pd@Z‐P/b CMR. Data are presented as mean ± SD. n = 5 per group. A maximum single deviation of the data from the mean within 3% is considered acceptable.

As shown in **Figure** **8**a, the Pd@Z‐P/b CMR exhibited excellent initial catalytic performance toward highly concentrated and deeply colored solutions of 2‐NA, MB, and MO, achieving conversion efficiencies of 95.3%, 99.9%, and 97.1%, respectively. However, the efficiency for 4‐NP declined to 50.3% after 6 h. This deactivation stems from the pronounced electronegativity of Pd@Z‐P (−33 mV, Table S3, Supporting Information), which facilitates strong electrostatic adsorption of positively charged 4‐AP, the reduction product of 4‐NP. Prolonged 4‐AP accumulation progressively blocks the active sites (Figure 6f), causing catalyst poisoning. In contrast, the reduction product of 4‐NA, 4‐PD (pK_a_ 6.0), does not protonate under alkaline conditions and is more weakly adsorbed than 4‐AP (pK_a_ 3.3). This difference, arising from their distinct conjugate acid pK_a_ values, allows 4‐PD to readily desorb, maintaining a dynamic equilibrium that ensures long‐term CMR stability. Similarly, the catalytic efficiency for 2‐NA conversion decreased to 68.2% after 12 h. This deactivation is probably due to the lower pK_a_ of 2‐NA (9.71) compared with that of 4‐NA (10.30),^[^ ^47^ ^]^ which promotes protonation and adsorption. Notably, the performance for MO was suboptimal, with the efficiency dropping to 85.7% after 24 h. This results from the room‐temperature precipitation of high‐concentration MO as orange crystals, which gradually block microchannels and impair reactor function.

These results demonstrate that the Pd@Z‐P/b CMR has relatively limited catalytic performance toward 2‐NA, 4‐NP, and MO. The electronegativity of the reactants is a key factor in catalyst deactivation. Therefore, a cationic dye MB solution was selected to evaluate the catalytic stability and durability. As depicted in Figure 8b, a 1.0 g L^−1^ MB solution and a 0.375 mol L^−1^ NaBH_4_ solution were continuously flowed through the CMR via a three‐way connector at a flow rate of 0.17 mL min^−1^. UV–vis spectroscopy showed that the MB absorption peak at 664 nm disappeared, and the solution turned from dark blue to colorless after the reaction. Moreover, during continuous 5‐d operation of the Pd@Z‐P/b CMR, the catalytic conversion efficiency for MB remained consistently above 97.5%. The turnover frequency (TOF) of the Pd@Z‐P/b CMR for 4‐NA and MB reached 1237.2 and 2213.4 h^−1^, respectively, outperforming previously reported systems (Table S4, Supporting Information). This clearly demonstrates the superior catalytic performance and high selectivity of the Pd@Z‐P/b CMR, which enables gram‐level processing of organic pollutants with minimal catalyst loading, surpassing most reported CMRs (Figure 8c). The exceptional stability and efficiency at high MB concentrations represent a significant advance in CMR design.

### 2.6 Regeneration and Practical Application in Environmental Water

Renewable performance is an essential requirement for the industrial application of catalysts, as it directly impacts cost control, operational efficiency, and environmental protection. Enhancing the stability, regeneration capability, and antipoisoning properties of catalysts can substantially improve their reusability, thereby promoting the sustainability of industrial processes. The renewable performance of the Pd@Z‐P/b CMR was evaluated by assessing its regeneration potential after being used in the catalytic process described in Section 3.4.

First, the used Pd@Z‐P/b CMR was thoroughly washed with deionized water at a flow rate of 0.17 mL min^−1^, and then a saturated 4‐NA solution was introduced at the same flow rate. As shown in Figure 8d, the catalytic efficiency of the reused Pd@Z‐P/b CMR recovered to over 99%, indicating that the active sites were successfully re‐exposed after washing. After 48 h of continuous operation, the catalytic efficiency of the Pd@Z‐P/b CMR remained above 98%. No signs of catalyst deactivation were observed compared with the results presented in Figure 8a. Subsequently, after washing the Pd@Z‐P/b CMR, a 2.5 g L^−1^ MO solution was introduced. The Pd@Z‐P/b CMR still exhibited excellent catalytic activity, achieving a conversion efficiency of 97%. These results allow us to conclude that the primary cause of the reduced catalytic efficiency of the Pd@Z‐P/b CMR is the adsorption of reduction products onto the catalyst active sites, rather than the loss of Pd SACs. This finding highlights the exceptional regenerability and sustainable utilization potential of the Pd@Z‐P/b CMR.

The catalytic performance and durability of the Pd@Z‐P/b CMR were evaluated using an environmental water sample. Specifically, water from the Fengjia River was selected as the experimental water source owing to its relatively turbid water quality and high concentrations of inorganic and organic substances (Figure S19, Supporting Information), which could potentially interfere with the normal operation of the CMR. The total organic carbon (TOC) and inorganic carbon (IC) contents in a slightly clearer water sample collected from the surface layer were quantified using a TOC analyzer, yielding concentrations of 11.06 mg·L^−1^ and 11.89 mg·L^−1^, respectively (Table S5, Supporting Information). Furthermore, the average particle size of microparticles in the water sample was determined to be 224.2 nm (Figure S20, Supporting Information).

**Figure 9:**
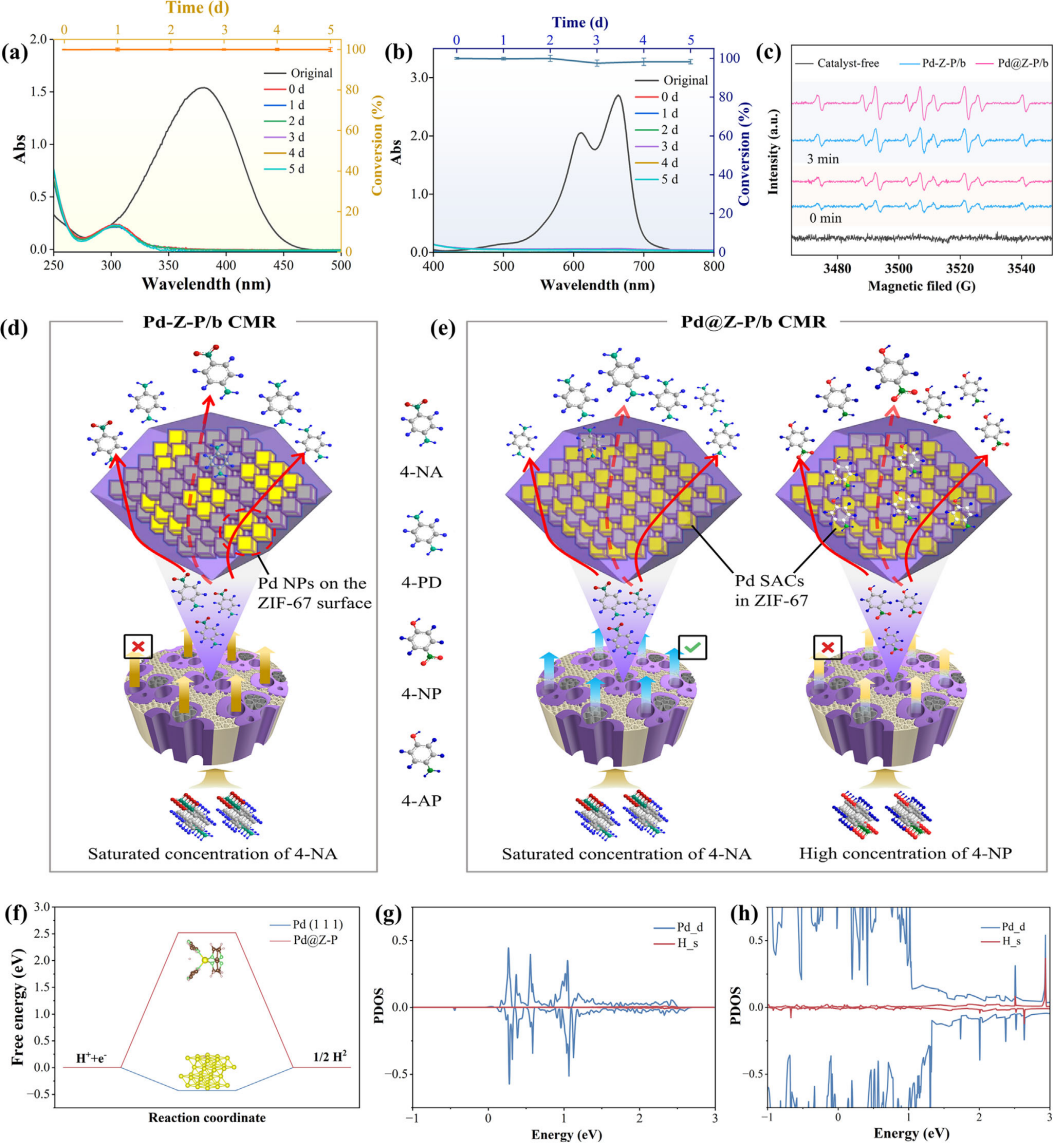
Practical applications for environmental water treatment: catalytic hydrogenation of a) 4‐nitroaniline (4‐NA) and b) methylene blue (MB). Data are presented as mean ± SD. n = 5 per group. A maximum single deviation of the data from the mean within 3% is considered acceptable. c) Representative EPR spectra of DMPO–H adducts. Schematic of d) Pd‐Z‐P/b CMR and e) Pd@Z‐P/b CMR in the presence of high concentration (reaching the gram level) of reactants. f) Hydrogen adsorption behavior of the Pd@Z‐P catalyst and Pd (111). Projected density of states for g) the Pd@Z‐P catalyst and h) Pd (111).

The UV–vis analysis of 4‐NA and NaBH_4_ dissolved in river water revealed a distinct peak at 380 nm (**Figure** **9**a), indicating the stability of the 4‐NA/NaBH_4_ system in complex, turbid environments. Upon flowing through the Pd@Z‐P/b CMR, a new peak emerged at 300 nm, confirming the formation of 4‐PD. The microreactor achieved >99% initial conversion efficiency and maintained near‐quantitative efficiency (>99%) after 5 days of continuous operation. Similarly, the dissolution of MB and NaBH_4_ in river water yielded a characteristic 664 nm peak (Figure 9b), demonstrating the structural integrity of MB. Remarkably, after passing through the Pd@Z‐P/b CMR, the MB peak disappeared completely. The conversion initially reached 99% and remained at 98.3% after 5 d, a performance comparable to that in deionized water. Critically, the Pd@Z‐P/b CMR considerably outperforms previously reported Ag/bamboo and Pd‐TiO_2_/bamboo microreactors for the treatment of cloudy Fengjia River water. To our knowledge, this is the first CMR to achieve continuous, gram‐level organic pollutant treatment in environmental water. The presence of diverse inorganic and organic substances did not cause significant interference. In conclusion, the Pd@Z‐P/b CMR exhibits exceptional performance in continuous‐flow hydrogenation in both deionized water and complex natural water samples.

### 2.7 Mechanism of Enhanced Catalytic Hydrogenation

Although catalytically inert, the PDA‐modified bamboo support provides an ideal platform for catalyst immobilization owing to its vascular microstructure. The naturally elongated microchannels offer an ultrahigh surface area‐to‐volume ratio (>30000 m^2^·m^−3^), enabling efficient catalyst anchoring and enhanced mass transfer—directly meeting the design criteria for high‐performance flow CMRs.^[^ ^6^, ^7^ ^]^ Previous studies indicate that the reactant/NaBH_4_ mixture undergoes a defined sequence within these microchannels: (1) reactant adsorption; (2) H^•^ generation from NaBH_4_/H_2_O followed by hydride formation (catalyst‐H); (3) hydrogen transfer; and (4) product desorption.^[^ ^6^, ^50^ ^]^ To further understand the reaction mechanism, an EPR measurements were conducted using 5,5‐dimethylpyrroline N‐oxide (DMPO) as a spin trapper to detect the presence of H^•^ species (Figure 9c). No obvious EPR signals were detected in the absence of a catalyst; however, the signal intensity increased at t = 0 and 3 min, demonstrating the catalyst's role in accelerating charge transfer to produce active species. It is worth noting that the signal peaks in the presence of the Pd@Z‐P catalyst were more pronounced than those with Pd‐Z‐P, which can be attributed to the superior electron transfer kinetics in the SAC configuration compared with nanoparticle‐based systems.^[^ ^19^ ^]^ The optimal signal intensity directly aligns with the superior catalytic performance across all tested CMRs.

Meanwhile, in the Pd‐Z‐P/b CMR, Pd nanoparticles are predominantly anchored on the surface of ZIF‐67 (Figure 9d). When a saturated 4‐NA solution flows through the Pd‐coated ZIF‐67 catalyst within the microchannels of bamboo (red dotted arrow), the catalytic hydrogenation reaction is restricted to the catalyst surface (red solid arrow). Despite the Pd loading being nearly three times higher than that in the Pd@Z‐P/b CMR, complete catalytic reduction of the saturated 4‐NA solution is unattainable owing to the limited number of active sites available for the reaction. Conversely, in the Pd@Z‐P catalyst, the catalytic hydrogenation reaction is not restricted to its surface but can also occur deep inside its structure (Figure 9e). Specifically, the high specific surface area (>1800 m^2^·g^−1^) and abundant nanopores of ZIF‐67 offer optimal conditions for the anchoring and uniform dispersion of Pd SACs. When a saturated 4‐NA solution infiltrates the ZIF‐67 framework, it markedly enhances the contact accessibility between the catalyst and reactants, thereby enabling the efficient catalytic hydrogenation of high‐concentration 4‐NA. In addition, N coordination in ZIF‐67 tailors the electronic structure of Pd SACs, inducing a higher positive charge that enhances electron affinity and catalytic activity (Figures 4 and 5). Concurrently, nanoporous confinement favors high‐electronegativity configurations (Table S3, Supporting Information), where support interactions reduce Pd electron density, thereby optimizing reactant adsorption and reaction kinetics. However, this CMR has certain limitations and is not applicable to all scenarios. If a strong electrostatic adsorption effect exists between the reaction product and the catalyst, preventing the reaction product from spontaneously desorbing from the catalytic active site, prolonged accumulation will eventually lead to catalyst deactivation (Figure 9e, sample 4‐NP).

Figure 9f compares the hydrogen adsorption behaviors of the Pd@Z‐P catalyst and Pd (111). The calculated hydrogen adsorption free energy on the Pd@Z‐P catalyst is as high as 2.52 eV, indicating extremely weak H* binding, whereas Pd (111) exhibits a much lower value of −0.43 eV, suggesting strong hydrogen affinity.^[^ ^51^, ^52^, ^53^ ^]^ The projected density of states further elucidates this difference: in the Pd@Z‐P system (Figure 9g), the Pd d states and H s states show almost no overlap near the Fermi level, implying negligible orbital hybridization and thus very weak Pd─H interaction. In contrast, for Pd (111) (Figure 9h), considerable overlap and hybridization between Pd d and H s orbitals are observed around the Fermi level, which stabilizes Pd─H bonding and accounts for the stronger H adsorption. These results demonstrate that the unique local coordination environment of the Pd@Z‐P catalyst effectively suppresses Pd─H orbital coupling, resulting in weaker hydrogen adsorption and distinct catalytic properties compared with metallic Pd (111). Beyond exceptional catalytic performance, the Pd@Z‐P/b CMR offers dual advantages that provide a viable route toward cost‐efficient catalytic processing: a facile preparation protocol without carbonization and dramatically reduced noble‐metal consumption (particularly Pd).

## 3 Conclusion

This work demonstrates a facile and efficient room‐temperature strategy for synthesizing and stabilizing Pd SACs within ZIF‐67 crystals during the formation of ZIF‐67. Key to this approach is the flow‐implantation of ZIF‐67 precursors into PDA‐modified bamboo microchannels, utilizing preimmobilized Co^2+^ seeds within the PDA matrices to anchor Pd atoms. The resulting Pd@Z‐P/b CMR, featuring isolated Pd─N_4_ sites within the ZIF‐67 framework and an ultralow Pd loading of only 0.0014 wt.%, exhibits remarkably superior performance and robustness in continuous‐flow catalytic hydrogenation compared with its nanoparticle‐loaded counterpart. This exceptional performance is directly attributed to the maximized atomic utilization efficiency of Pd SACs. Specifically, the catalyst achieves 94.3% efficiency in the reduction of saturated 4‐NA over 10 d of continuous operation and 97.5% efficiency in the hydrogenation of gram‐level concentrations of MB over 5 d. The consistently exceptional catalytic performance, even with real environmental water samples, highlights the practical potential of the Pd@Z‐P/b CMR. Mechanistic insights reveal that the high surface area and porosity of ZIF‐67 are crucial for stabilizing Pd SACs, enhancing metal dispersion, adjusting the coordination environment, and ensuring their accessibility during catalysis. Furthermore, the incorporation of Pd SACs establishes an efficient electron transfer pathway and optimizes hydrogen binding energy, thereby enhancing the catalytic hydrogenation activity. This study presents not only a novel room‐temperature pathway for synthesizing highly effective metal SACs but also provides an effective and robust strategy for the continuous‐flow treatment of high‐concentration organic pollutants, demonstrating potential for practical environmental remediation applications.

## 4 Experimental Section

### Materials

Specimens measuring 10 mm in diameter and 100 mm in length (along the longitudinal growth direction) were collected from the internodes of naturally colored, four‐year‐old moso bamboo (*Phyllostachys edulis*) sourced from Anqing, Anhui Province, China. The following chemicals were procured from Aladdin (Shanghai, China): Pd(OAc)_2_, Co(NO_3_)_2_·6H_2_O, 2‐MI, NaBH_4_, NH_4_OH, dopamine hydrochloride, methanol, 4‐NA, 4‐NP, 2‐NA, MB, and MO. Tris‐(hydroxymethyl)‐aminomethane (Tris) was purchased from Sango Biotech (Shanghai, China). All reagents were used as received without further purification.

### Synthesis of the Pd@Z‐P/b CMR

In a typical procedure for immobilizing ZIF‐67‐confined Pd SACs in PDA‐modified bamboo microchannels, bamboo strips were sealed on their sides with heat‐shrink tubing to ensure that the liquid flowed exclusively along the bamboo growth direction. Subsequently, the two ends of the bamboo strips were securely connected to silicone tubes, thus establishing a flow‐assisted preparation system. First, the inner surface of the bamboo microchannels was chemically modified via NH_3_·H_2_O treatment to promote the uniform growth of the PDA matrices. Specifically, a 10 wt.% NH_3_·H_2_O solution was circulated through the microchannels at a flow rate of 1.66 mL min^−1^ using a peristaltic pump for 10 min, ensuring complete reaction with the inner walls of the microchannels. Subsequently, the microchannels were thoroughly rinsed with deionized water until the effluent pH reached neutrality (pH 7). Then, under ambient conditions, 0.546 g of Co(NO_3_)_2_·6H_2_O and 0.06 g of dopamine hydrochloride were completely dissolved in a Tris buffer solution (10 mmol L^−1^, pH 8.5). The resulting solution was then circulated through the NH_3_‐treated bamboo samples at a flow rate of 0.5 mL min^−1^ for 5 h. Finally, the samples were repeatedly washed with methanol, leading to the formation of Co^2+^‐crosslinked PDA/bamboo, denoted as Co‐PDA/b CMR.

Second, 1.232 g of 2‐MI and 33 mg of Pd(OAc)_2_ were sequentially added to a methanol solution under stirring. After complete dissolution, the resultant mixed solution was continuously circulated into the Co‐PDA/b CMR system at 0.5 mL min^−1^ for 15 h. Subsequently, the sample was sequentially washed with methanol and deionized water until the effluent reached neutrality. Finally, the obtained sample was dried at 50 °C for 12 h, yielding a bamboo‐based CMR loaded with ZIF‐67‐confined Pd SACs (the Pd@Z‐P/b CMR).

As a control experiment, Z‐P/b CMR without incorporating the Pd(OAc)_2_ precursor solution was also prepared, while ensuring that all other reaction conditions were identical to those described above. Moreover, by using 10 and 30 mm Co(NO_3_)_2_·6H_2_O solutions and systematically adjusting the molar ratio of 2‐MI to the Co source, both the crystal and pore sizes of ZIF‐67 were effectively controlled. Accordingly, two sets of control samples were prepared, i.e., Pd@Z(10)‐P/b CMR and Pd@Z(30)‐P/b CMR. Furthermore, by varying the duration of the Pd loading step to 5 and 10 h, two additional samples with differing Pd loading amounts were prepared and named Pd(5)@Z‐P/b CMR and Pd(10)@Z‐P/b CMR, respectively. All other experimental conditions were as described above.

### Synthesis of the Pd‐Z‐P/b CMR

The procedure involves three main steps. First, the Co‐PDA/b CMR was prepared in accordance with Section 4.2. The difference lies in the second step: 1.232 g of 2‐MI was added to a methanol solution under continuous stirring. After complete dissolution, the resulting mixture was uniformly introduced into the Co‐PDA/b CMR at a flow rate of 0.5 mL min^−1^ for 15 h of cyclic reaction. Subsequently, 33 mg of Pd(OAc)_2_ was added to a separate methanol solution. After complete dissolution, the solution was introduced into the system under identical flow rate conditions for an additional 15 h of cyclic reaction. The samples were cleaned and dried as described in Section 4.2. The fabricated bamboo‐based CMR featuring nanosized Pd particles loaded onto the surface of the ZIF‐67/PDA layer was denoted as Pd‐Z‐P/b CMR.

### Characterization

The separated powder catalyst used for detailed structural characterization (Pd@Z‐P) was obtained via ultrasonic treatment within the Pd@Z‐P/b CMR microchannels.^[^ ^3^, ^24^ ^]^ The morphology of the bamboo‐based CMRs was characterized via field‐emission SEM (Gemini SEM 560, Germany). The elemental distribution was analyzed using EDS (Bruker Xflash 6130). The microscopic structure of the catalysts was observed using TEM conducted on a field‐emission transmission electron microscope (FEI Talos F200S, USA). The existence of Pd SACs was investigated using AC‐HAADF‐STEM (JEM‐ARM300F, Japan). The porosity was determined via mercury intrusion using a MicroActive AutoPore V 9600 (Micromeritics, USA). The pore size variation was determined via BET test (Quantachrome Autosorb‐1, USA). The XRD patterns were collected using a Rigaku Ultima IV X‐ray diffractometer with Cu K*α* radiation (Bruker D8 Advance, Germany). XPS spectra were acquired using a Thermo ESCALAB 250Xi spectrometer (Thermo Scientific, Waltham, MA, USA) with an Al K*α* X‐ray source. The TOC and IC contents in the water sample were quantified using a TOC analyzer (Multin/c3100, JENA, Germany). The detection of H^•^ species was performed in a batch fashion. EPR spectra were recorded using a Bruker EMX PLUS spectrometer (Germany). To quantify the Pd loading in the CMR, completely dried samples were cut into small pieces, dissolved in aqua regia, and analyzed via ICP‐MS (Agilent 7700). Zeta potential was measured by dispersing the catalysts in methanol and adjusting the pH to 14 with NaOH using a Malvern Zetasizer Nano 2590 (UK). XAFS for Pd was measured at the BL14W1 beamlines at the Shanghai Synchrotron Radiation Facility (Shanghai, China). The XAFS spectra were recorded at room temperature using a four‐channel silicon drift detector (Bruker 5040). Pd K‐edge EXAFS spectra were recorded in fluorescence mode. Negligible changes in the line shape and peak position of the Pd K‐edge XANES spectra were observed between two scans for a specific sample. The XAFS spectra of PdO and Pd foil as standard samples were recorded in transmission mode. The spectra were processed and analyzed using the software codes Athena and Artemis. Furthermore, the assignment of the Pd‐N_4_ configuration was collectively supported by EXAFS fitting, the absence of Pd‐O peaks in XPS O 1s spectra, and DFT modeling.

### Catalytic Continuous‐Flow Hydrogenation Performance

The catalytic hydrogenation performance of the Pd@Z‐P/b CMR was evaluated using the reduction of 4‐NA with NaBH_4_ as a model reaction. NaBH_4_ was a mild, fast‐acting reductant with a simple work‐up. This reaction proceeds at room temperature without the formation of byproducts and can be visually monitored through color change and precisely tracked using a UV–vis spectrophotometer (Shimadzu UV‐2550, Kyoto, Japan).

In a standard procedure, a freshly prepared 0.8 g L^−1^ 4‐NA solution (25 °C, saturated concentration) and a 0.375 M NaBH_4_ solution were continuously introduced into the bamboo‐based CMR at a controlled flow rate of 0.17 mL min^−1^. The resulting product solution was collected from the CMR outlet and subsequently diluted 12‐fold with deionized water. The product conversion was quantified using UV–vis spectrophotometry at 380 nm, with a scanning range of 250–500 nm. The 4‐NA conversion efficiency was calculated using the equation: conversion efficiency = (A_0_ − A)/A_0_, where A_0_ and A represent the concentrations of 4‐NA in the influent and effluent solutions, respectively. To quantitatively investigate the catalytic efficiency, the TOF, defined as the number of moles of reactant converted per mole of catalyst per hour, was calculated using the equation TOF = n_react_/(n_cat_×t), where n_react_ and n_cat_ represent the number of moles of reacted 4‐NA and the Pd catalyst, respectively, and t is the time. The weight and internal volume of the bamboo‐based CMR were 4.5 g and 1 mL, respectively, the Pd amount was 14 mg kg^−1^ (sample number = 5), and the calculated residence time was 15–16 s. Each experiment was performed in five independent replicates. The mean value and standard deviation were used for plotting. A maximum single deviation of the data from the mean within 3% was considered acceptable. Statistical analysis and visualization were performed using Origin.

### Catalytic Activity in the Hydrogenation of Various High‐Concentration Organic Pollutants

To confirm the universality of the Pd@Z‐P/b CMR, a series of catalytic hydrogenation reactions was conducted under strictly controlled, identical experimental conditions using the following high‐concentration organic pollutants: 2‐NA (1.1 g L^−1^, 25 °C, saturation concentration), 4‐NP (0.5 g L^−1^), MB (1.0 g L^−1^), and MO (2.5 g L^−1^). Solutions of these organic compounds at the specified concentrations, together with a 0.375 M NaBH_4_ solution, were continuously flowed through the bamboo‐based CMR at a rate of 0.17 mL min^−1^. The product solutions were collected from the CMR outlet. The effluents of the 2‐NA and 4‐NP solutions in the Pd@Z‐P/b CMR were diluted 12‐fold with deionized water, whereas the effluents of MB and MO were diluted 24‐fold. Subsequently, UV–vis spectra were recorded to analyze the product conversions at 412, 400, 680, and 665, respectively.

### Catalytic Hydrogenation Activity in Real Environmental Water Samples

To investigate the robustness and durability of the Pd@Z‐P/b CMR, catalytic hydrogenation activity was assessed using real environmental water samples collected from the Fengjia River in Hangzhou, China, as the reaction medium with saturated 4‐NA and high‐concentration MB (1.0 g L^−1^). All reaction conditions were maintained as outlined in Section 4.5.

### Computational Details

All calculations were conducted within the spin‐polarized density functional theory framework, as implemented in the Vienna ab initio Simulation Package. Ion–electron interactions were treated using the projector augmented‐wave method. The generalized gradient approximation with the Perdew–Burke–Ernzerhof functional was used to describe the electronic exchange–correlation interactions, with a plane‐wave cutoff energy of 450 eV. Van der Waals interactions were considered to correct dispersion effects using Grimme's DFT‐D3 approach. The geometric optimization convergence threshold for atoms was set at 0.01 eV Å^−1^, with self‐consistent accuracy set to 10^−5^ eV. A Gamma‐centered 3 × 3 × 1 mesh was used to sample the first Brillouin zone.

The Pd─N_4_ structure was constructed by isolating the local coordination environment from ZIF‐67. Specifically, the representative Co─N_4_ coordination unit was extracted, and the central Co atom was substituted with Pd to form a Pd─N_4_ moiety. The surrounding carbon backbone was partially retained to preserve the chemical environment and structural rigidity of the ligand framework. This construction strategy, widely adopted in the theoretical modeling of SACs, ensures that the Pd site was embedded in a realistic N‐coordination environment while maintaining computational tractability. In experimental systems, Pd atoms were typically stabilized by N‐doped carbon matrices or MOF‐derived carbons, where the Pd─N_4_ unit represents the dominant local coordination environment. By deriving the Pd─N_4_ moiety directly from ZIF‐67, the model preserves the geometric and electronic properties observed experimentally, and removing the extended periodic framework enhances computational efficiency without sacrificing essential local chemistry. Therefore, the constructed Pd─N_4_ model can reasonably capture the bonding properties and catalytic behavior of Pd SACs embedded in N‐doped carbon supports.

For Pd models, the (111) facet consisting of four atomic layers was used. The bottom two layers were fixed to mimic bulk properties, whereas the remaining two layers and surface‐adsorbed species were fully relaxed. A vacuum space of up to 15 Å was employed to prevent interlayer interactions in the *z*‐direction. The free energy change (ΔG) of the elementary step was computed using ΔG = ΔE_DFT_ + ΔE_ZPE_ − TΔS + eU, where ΔE_DFT_ represents the change in DFT total energy for the species involved, T was the system temperature (298.15 K), and ΔE_ZPE_ and ΔS refer to the zero‐point energy change and entropy change, respectively. In the electrochemical steps of the computational hydrogen electrode model, the chemical potential of a pair of proton and electron can be correlated as µ(H+) + µ(e−) = 1/2 µ (H+) − eU, where the potential U is referenced to the standard hydrogen electrode.

## Conflict of Interest

The authors declare no conflict of interest.

## Supporting information



Supporting Information

## Data Availability

The data that support the findings of this study are available from the corresponding author upon reasonable request.
