# Supplementary material for: ZIF‐67‐Confined Pd Single‐Atom Catalysts Implanted Into Polydopamine‐Modified Bamboo Microchannels for Robust Continuous‐Flow Hydrogenation
Source: Adv Sci (Weinh). 2025 Dec 7;13(11):e13960. doi: 10.1002/advs.202513960 (PMC12931216; doi:10.1002/advs.202513960)
Supplement: Supplementary file 1 — Supporting Information [file ADVS-13-e13960-s001.docx]

**Supporting Information**

**ZIF-67-Confined Pd Single-Atom Catalysts Implanted into Polydopamine-Modified Bamboo Microchannels for Robust Continuous-Flow Hydrogenation**

Sisi Yao ^1,2,4^, Jiawei Han ^1^, Wenjun Zhang ^1^, Dengkang Guo ^1^, Shenjie Han ^3^, Yun Lu ^2^*, Jingpeng Li ^1^*

^1^ Engineering Technology Research Center for Building and Decorating Materials of Bamboo State Forestry Administration, China National Bamboo Research Center, Hangzhou 310012, China

^2^ Research Institute of Wood Industry, Chinese Academy of Forestry, Beijing 100091, China

^3^ Key Laboratory for Advanced Technology in Environmental Protection of Jiangsu Province, Yancheng Institute of Technology, Yancheng 224051, China

^4^ School of Chemistry and Chemical Engineering, Southeast University, Nanjing, Jiangsu 211189, China

* Corresponding author: Email: lijp@caf.ac.cn (J. Li); y.lu@caf.ac.cn (Y. Lu)


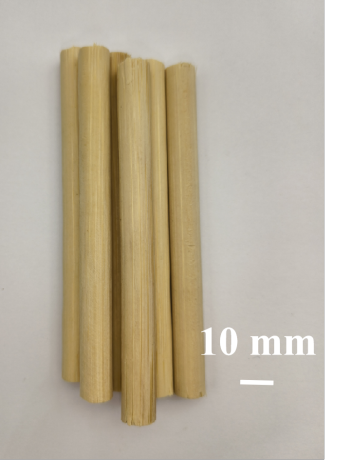


Figure S1. Digital image of the original bamboo specimens.


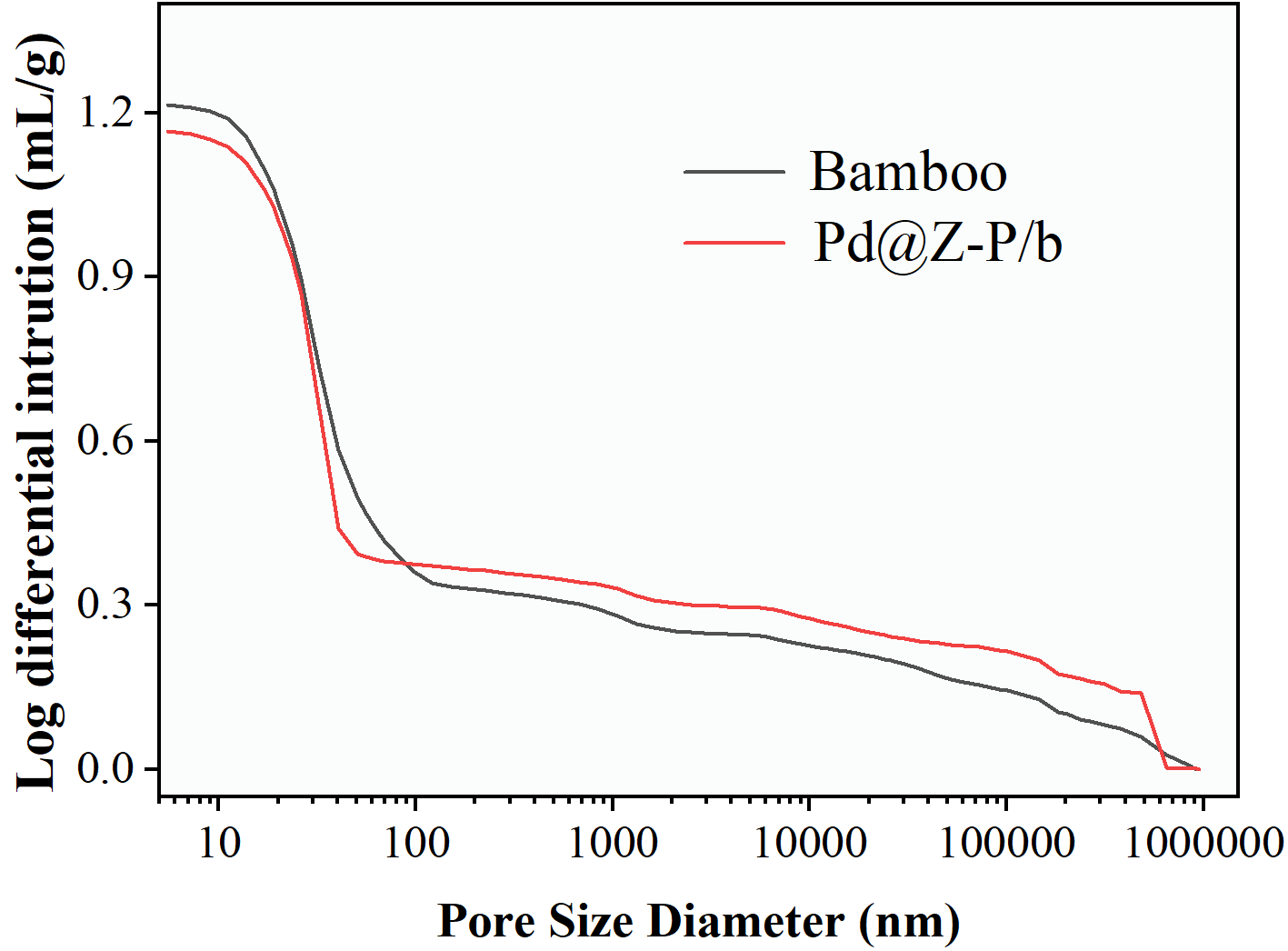


Figure S2. Cumulative intrusion as a function of pore diameter.


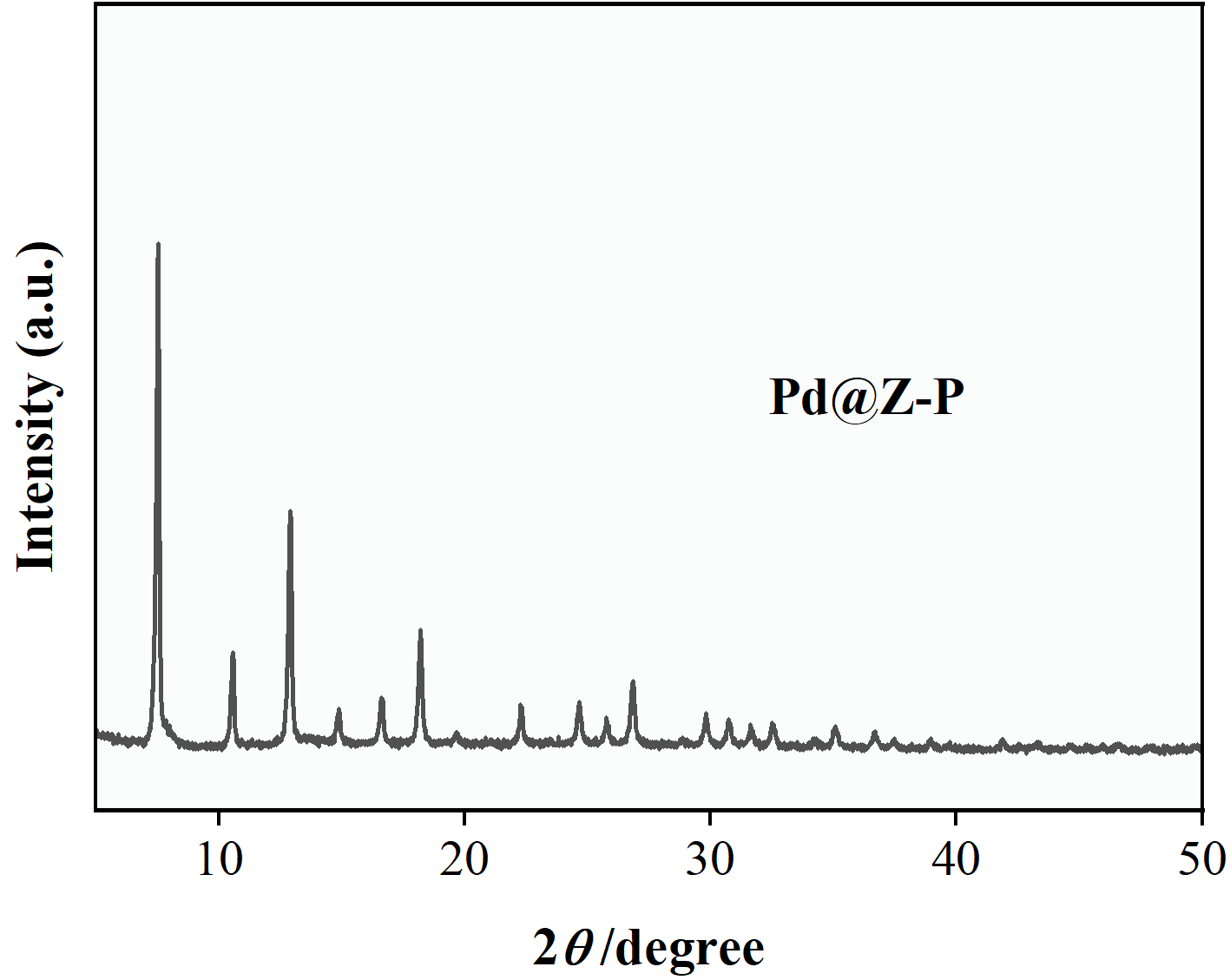


Figure S3. XRD pattern of the Pd@Z-P catalysts.


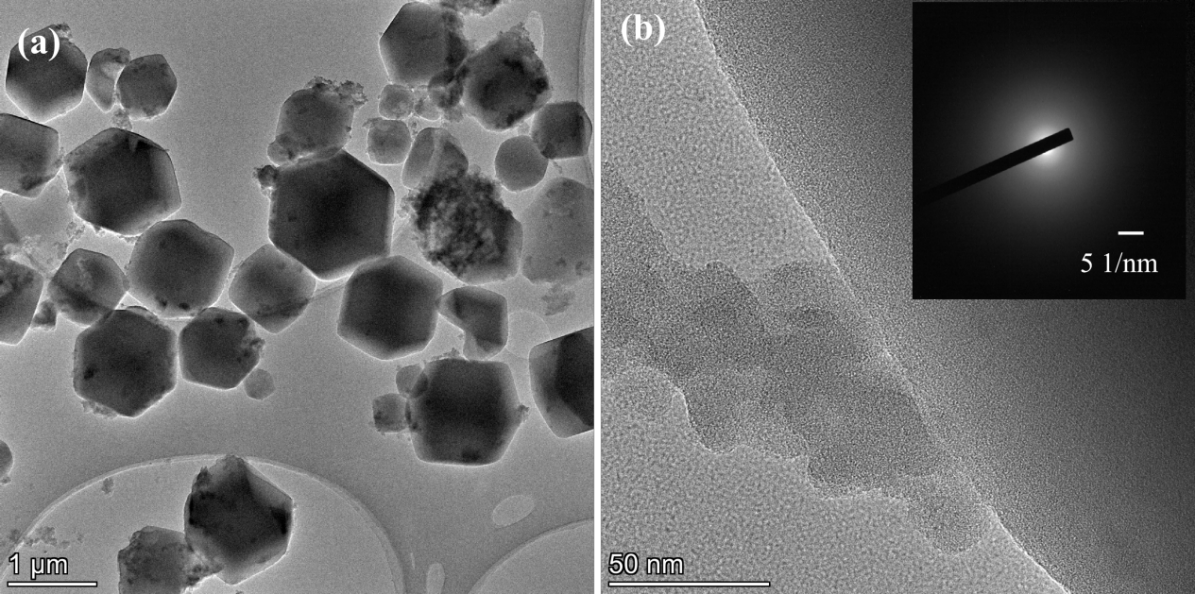


Figure S4. TEM images of the Pd@Z-P catalysts at various magnifications.


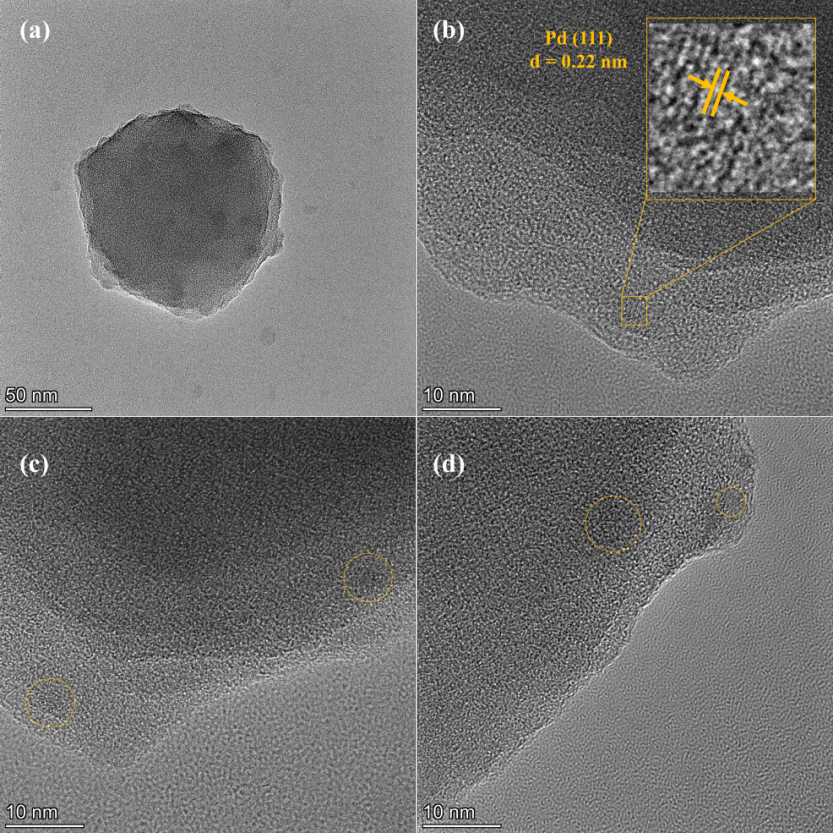


Figure S5. TEM images of the Pd-Z-P catalysts.


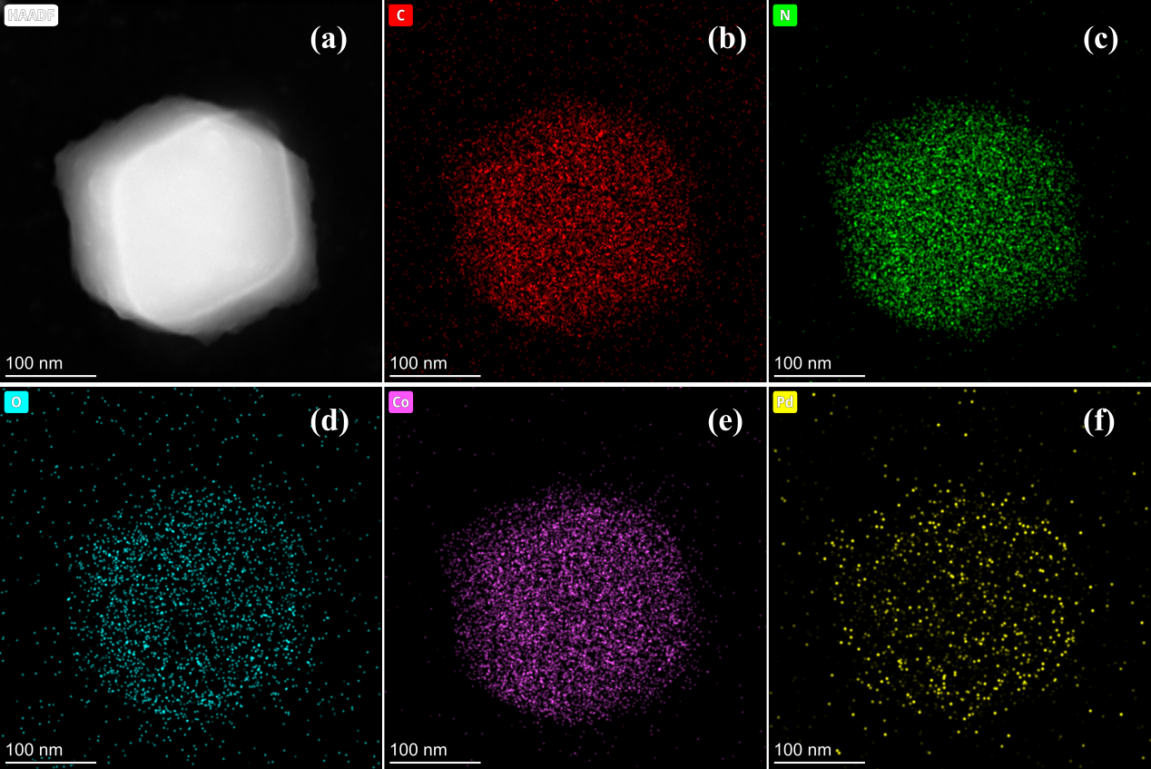


Figure S6. HAADF-STEM and EDS mapping results of Pd-Z-P catalysts.


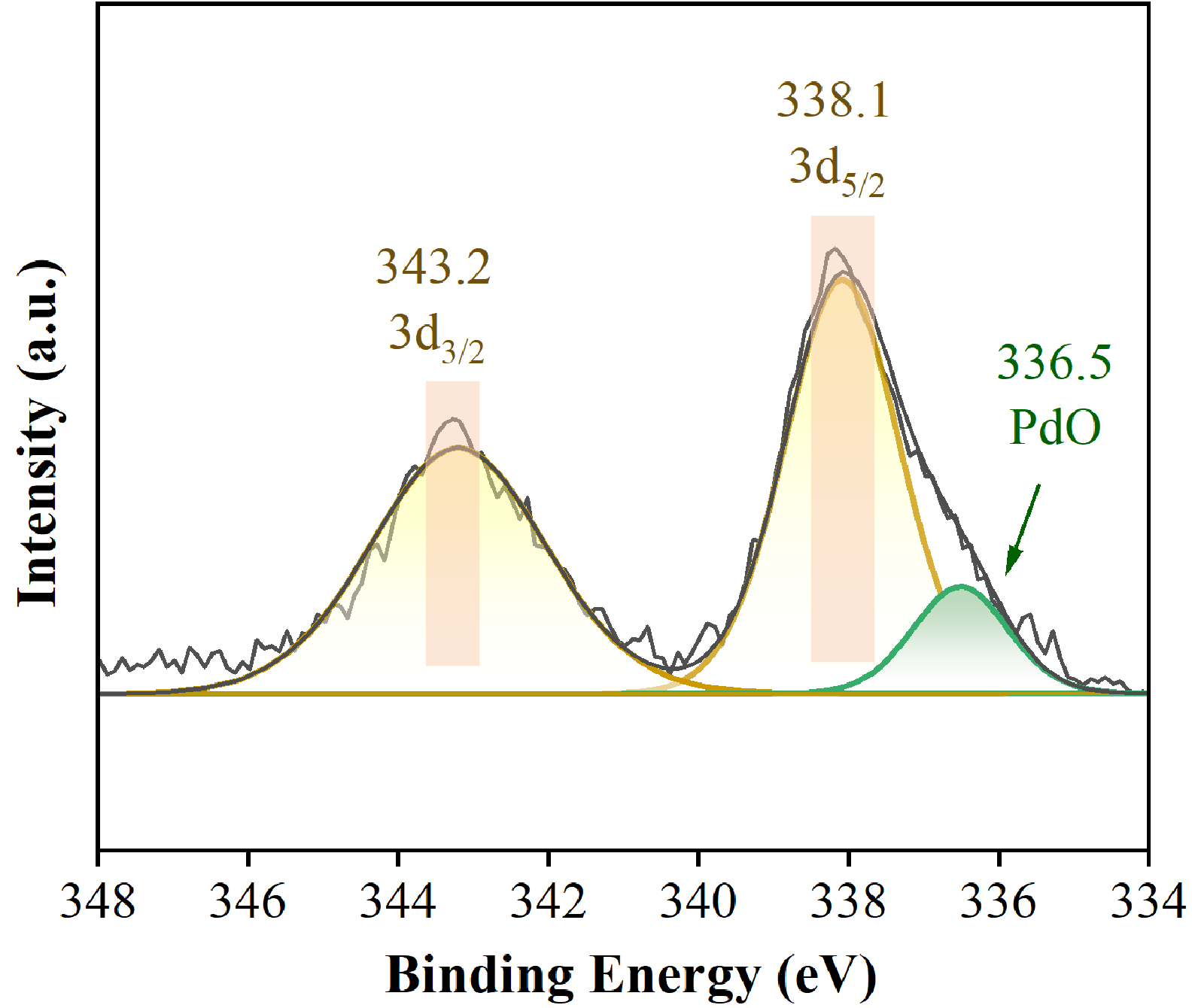


Figure S7. The Pd 3d spectrum of Pd-Z-P catalysts.


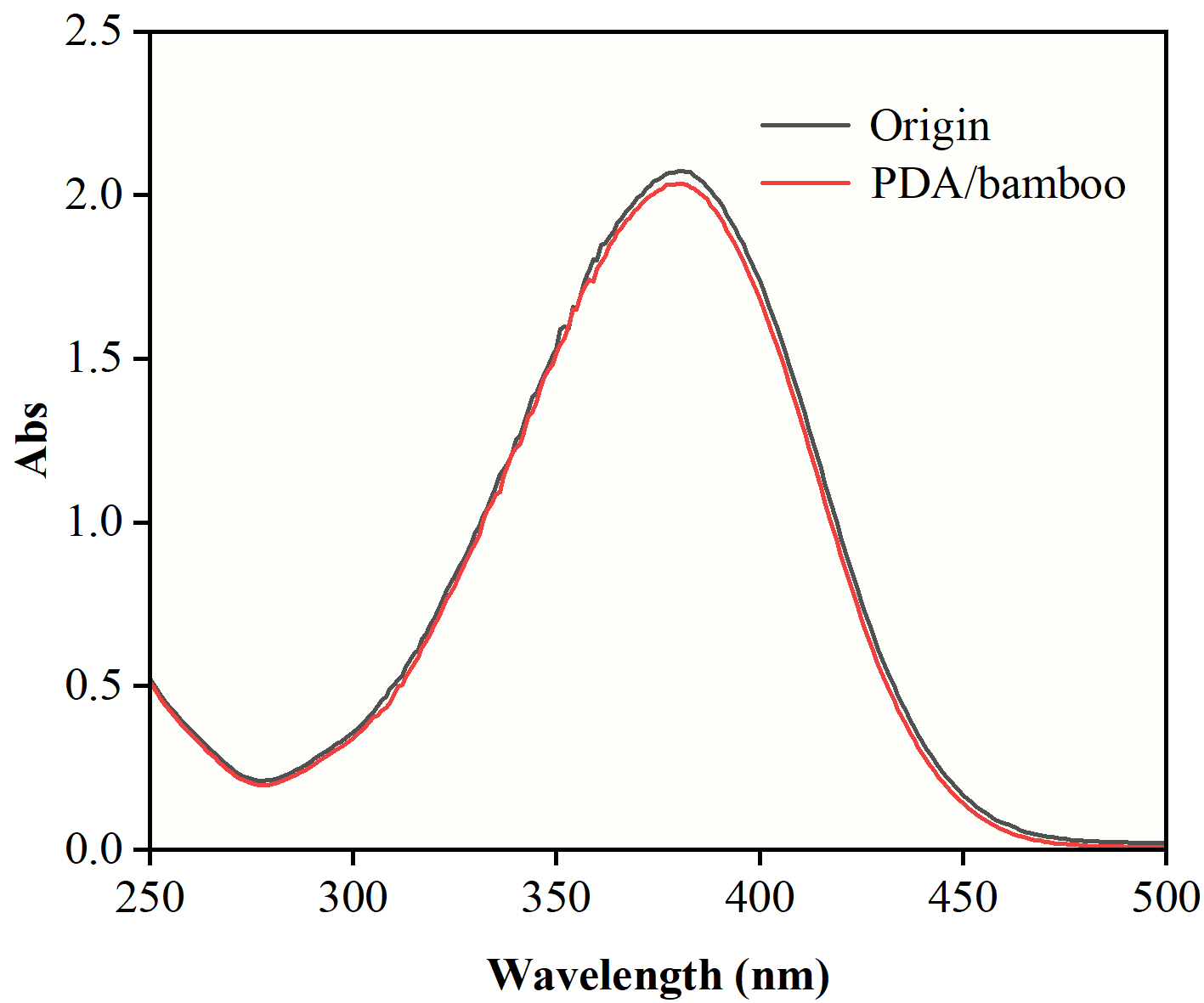


Figure S8. The catalytic performance of PDA/bamboo CMR.


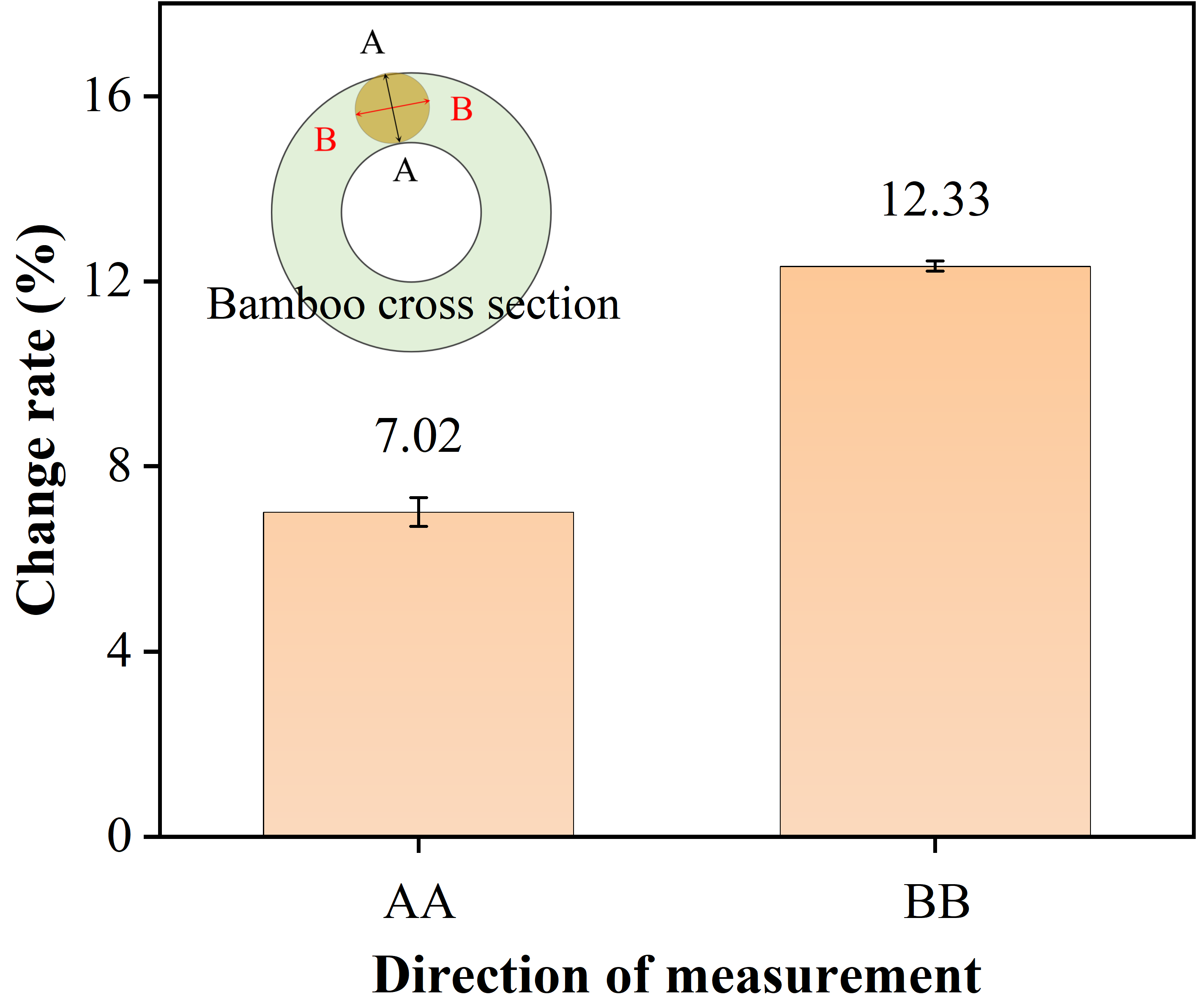


Figure S9. The dry shrinkage rate of the Pd@Z-P/b CMR in both directions along the diameter of bamboo stick at different run time. The inset is a test location diagram.


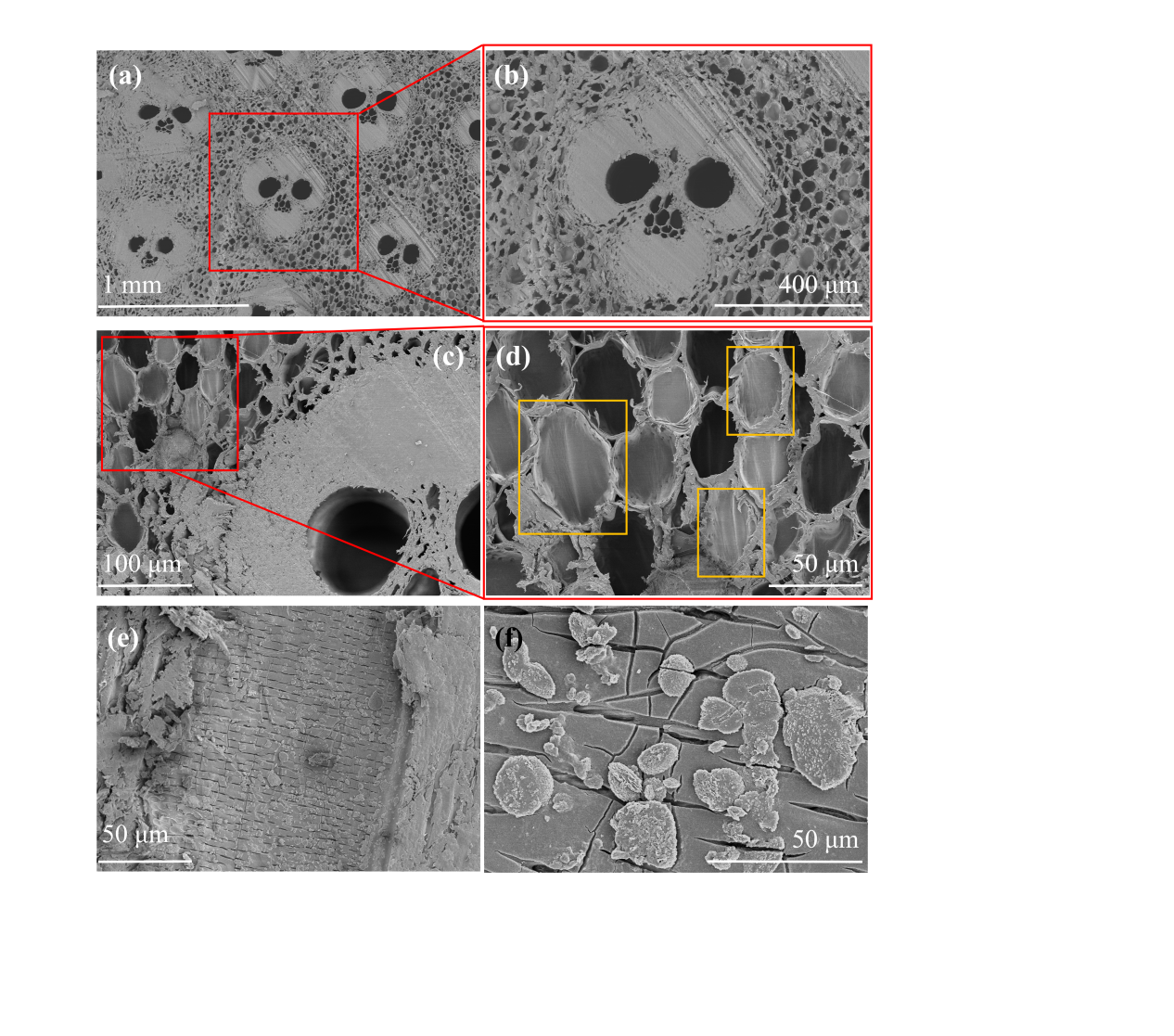


Figure S10. SEM of Pd@Z-P/b CMR after 10 d of continuous operation.


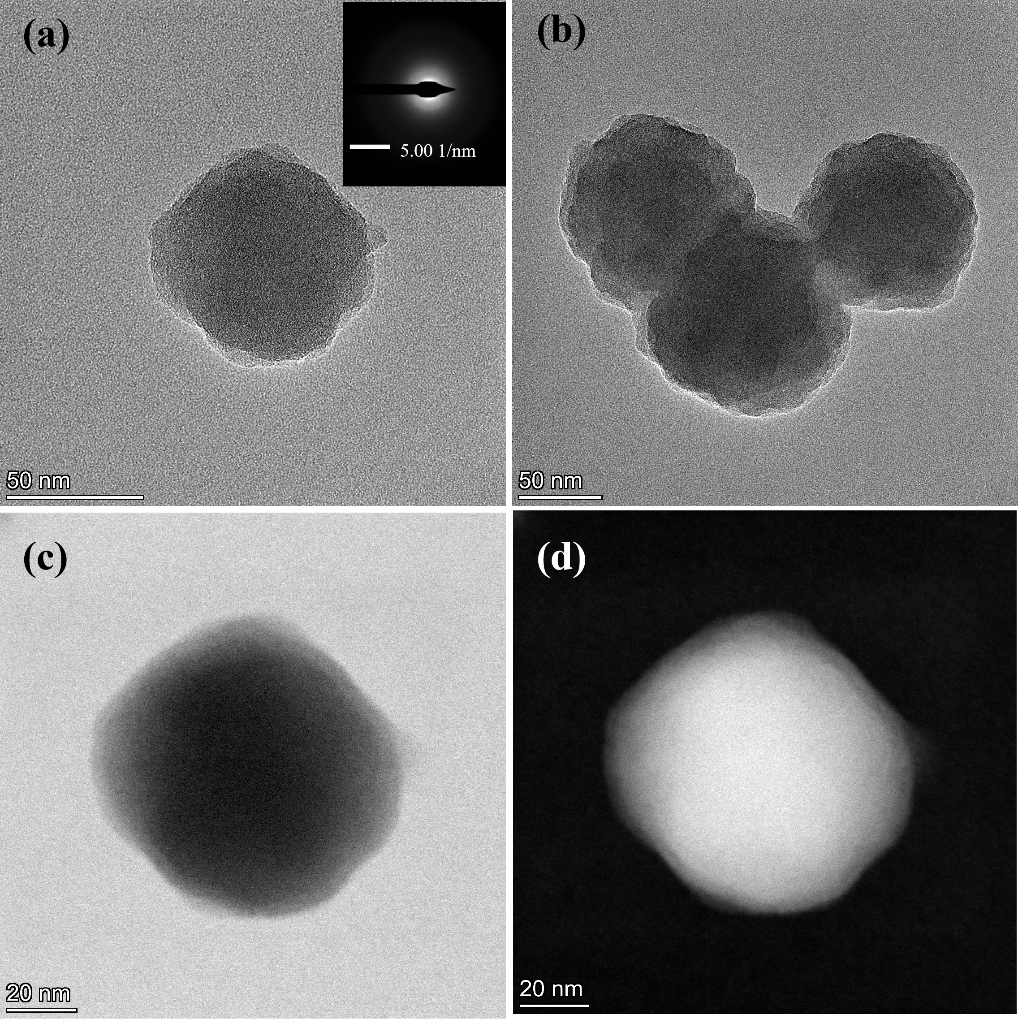


Figure S11. TEM of Pd@Z-P catalysts after 10 d of continuous operation.


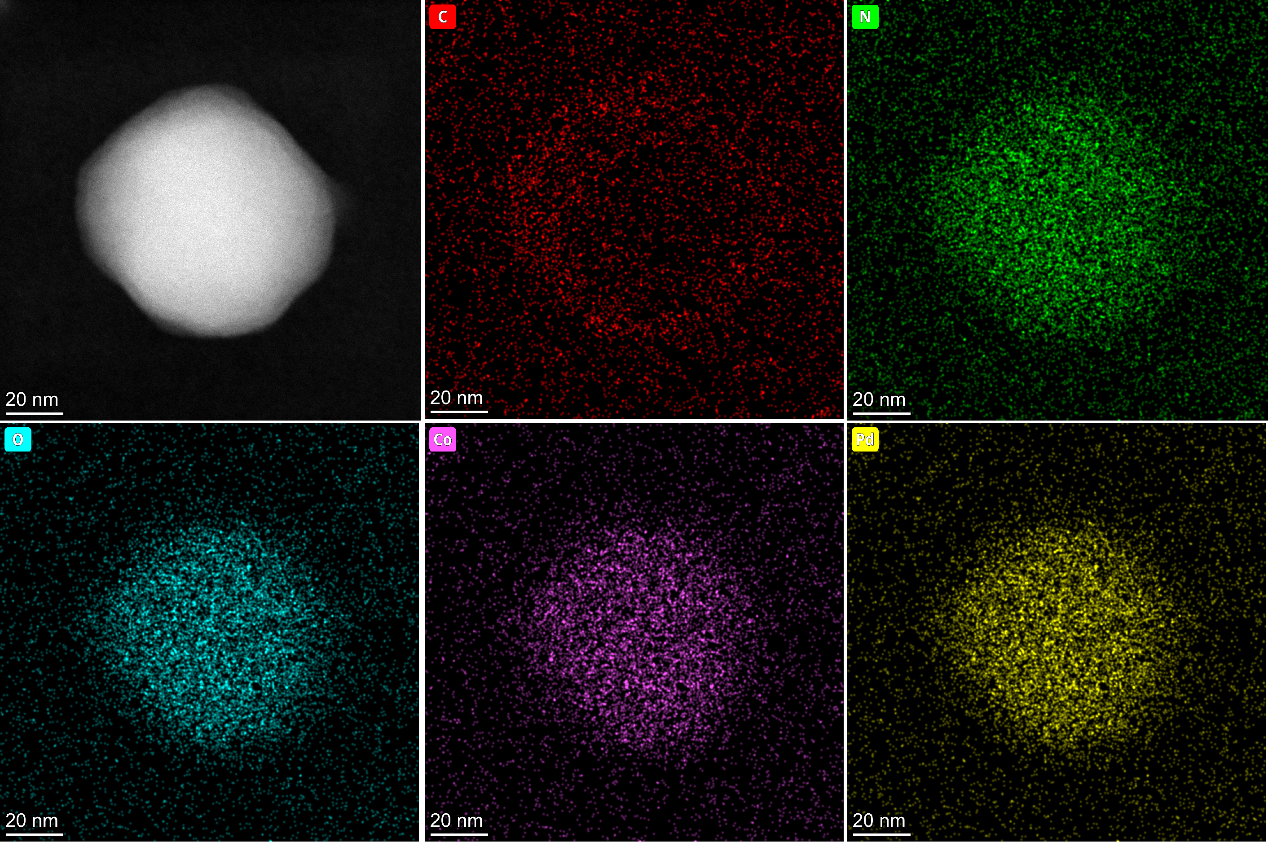


Figure S12. HAADF-STEM and EDS mapping results of Pd@Z-P catalysts after 10 d of continuous operation.


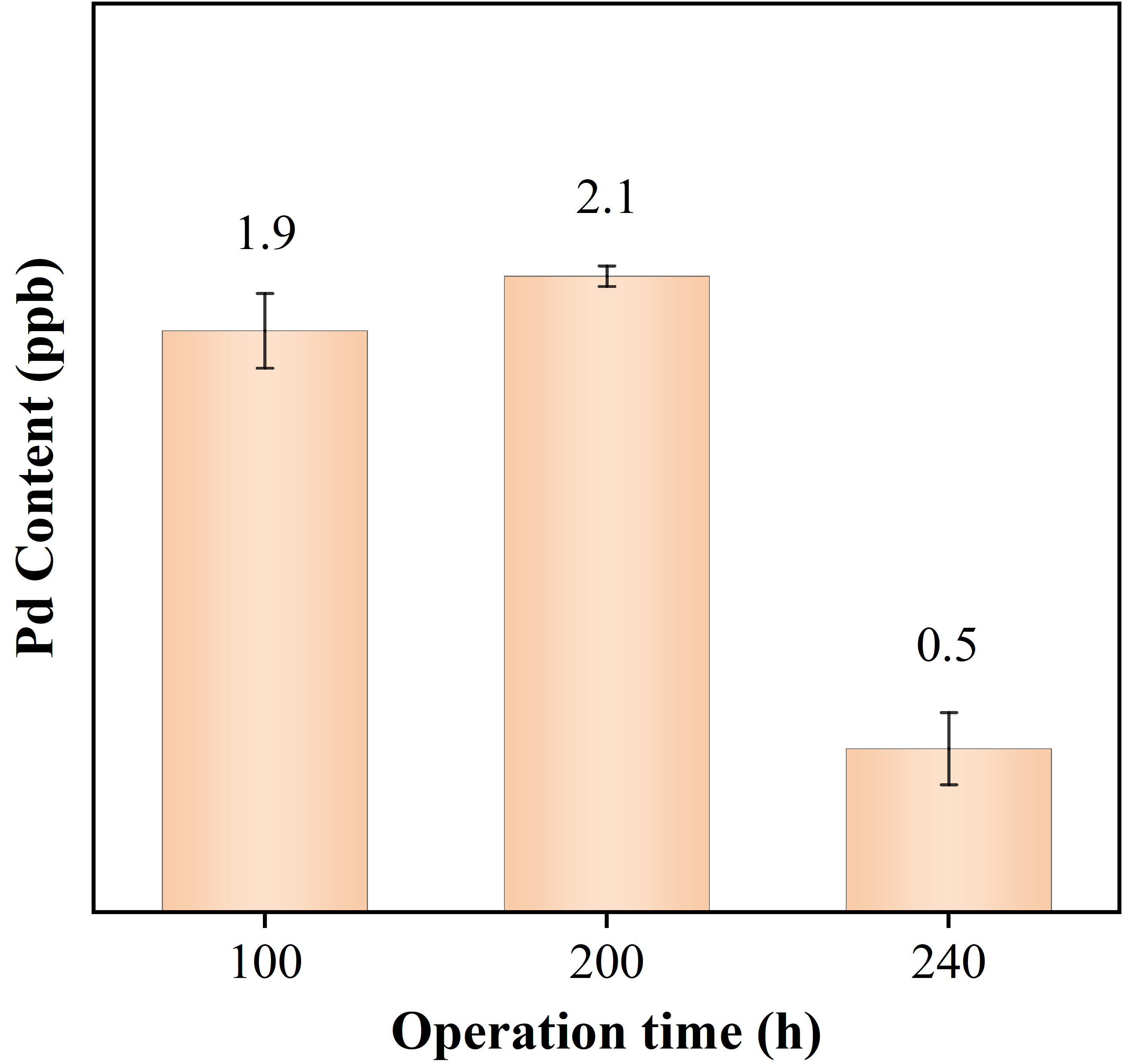


Figure S13. The content of Pd in the effluents within the entire effluent.


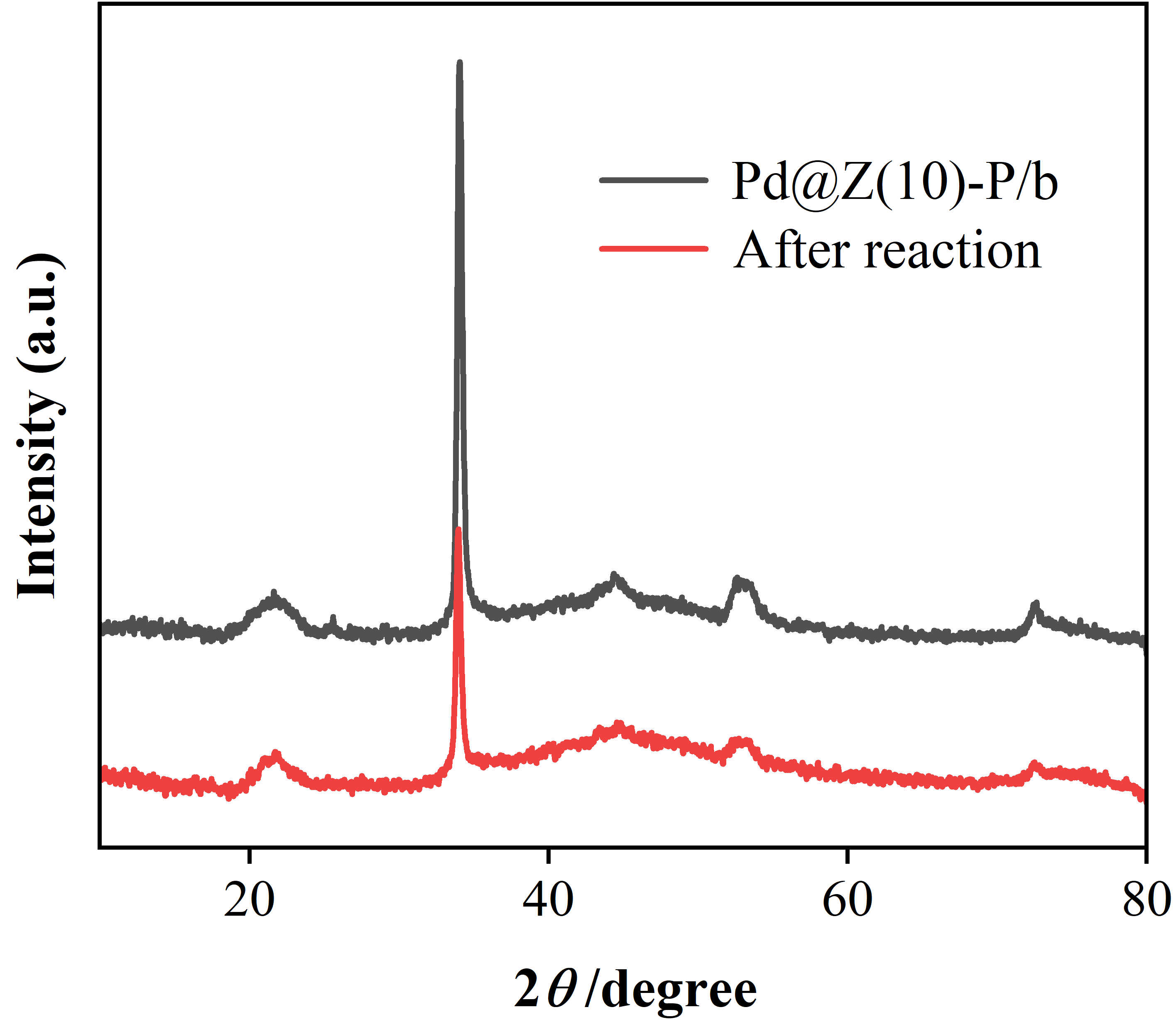


Figure S14. XRD spectra of Pd@Z-P/b CMR after 10 d of continuous operation.


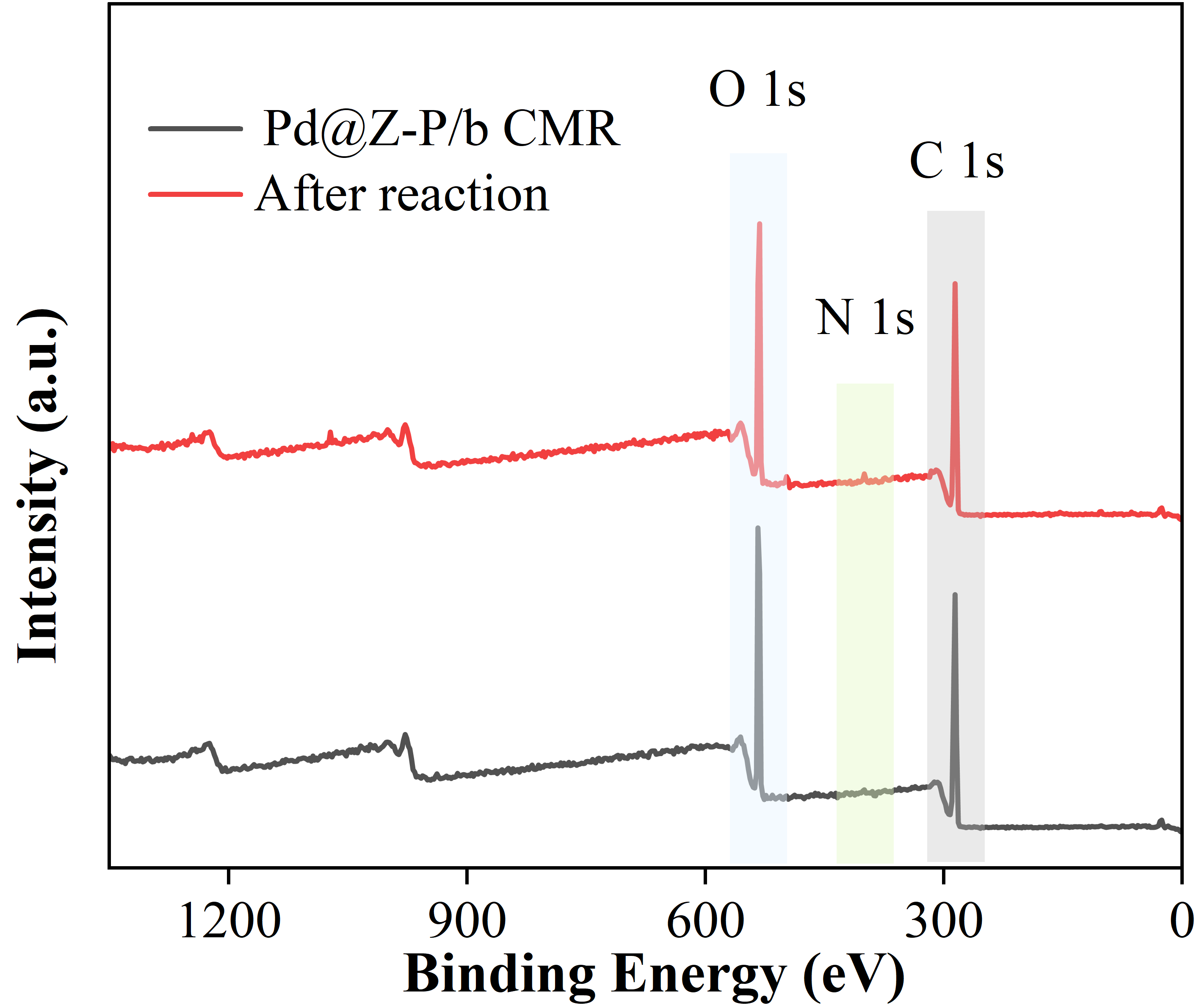


Figure S15. XPS spectra of Pd@Z-P/b CMR after 10 d of continuous operation.


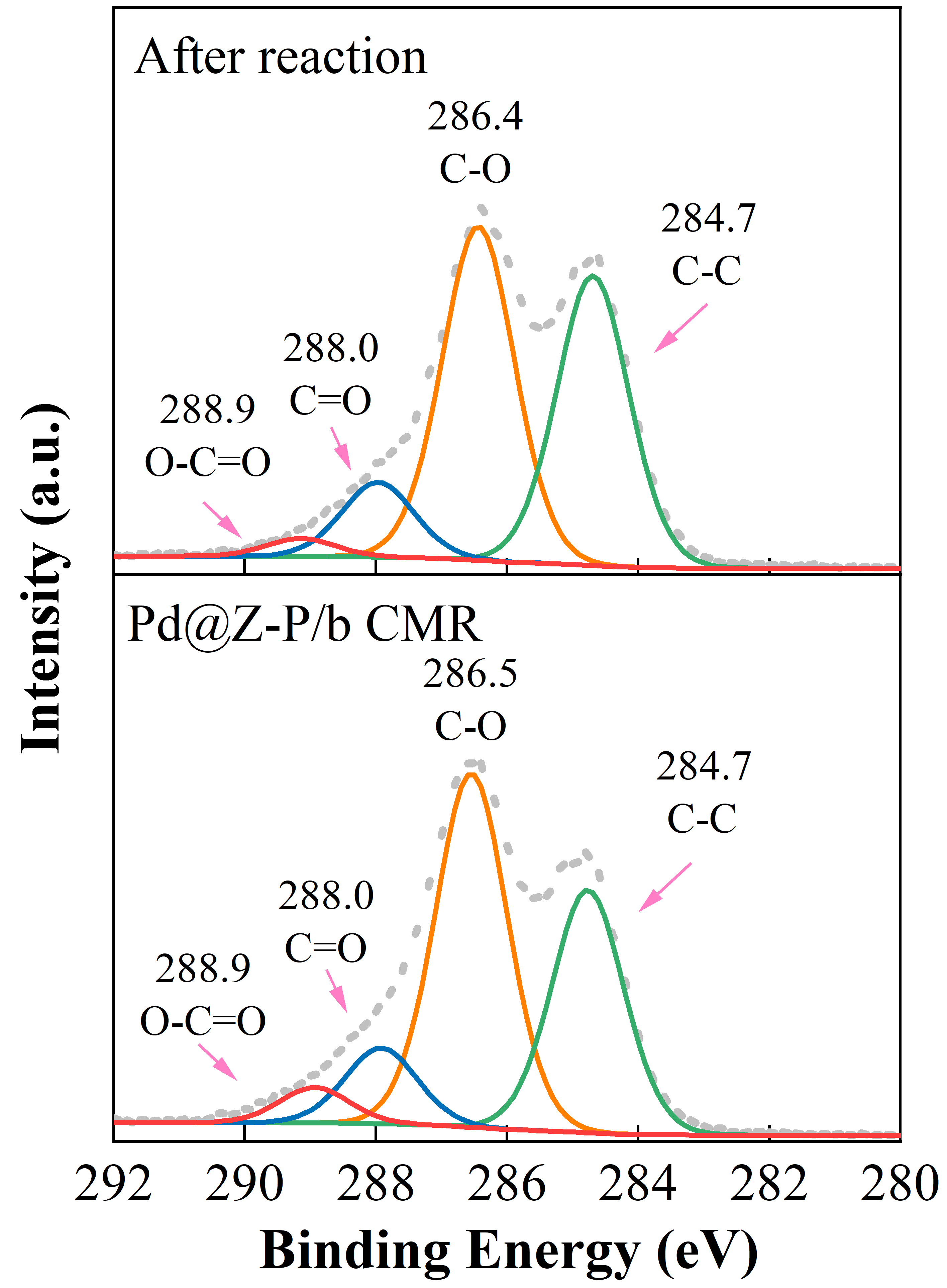


Figure S16. High resolution XPS spectrum for C 1s of Pd@Z-P/b CMR

before and after 10 d operation.


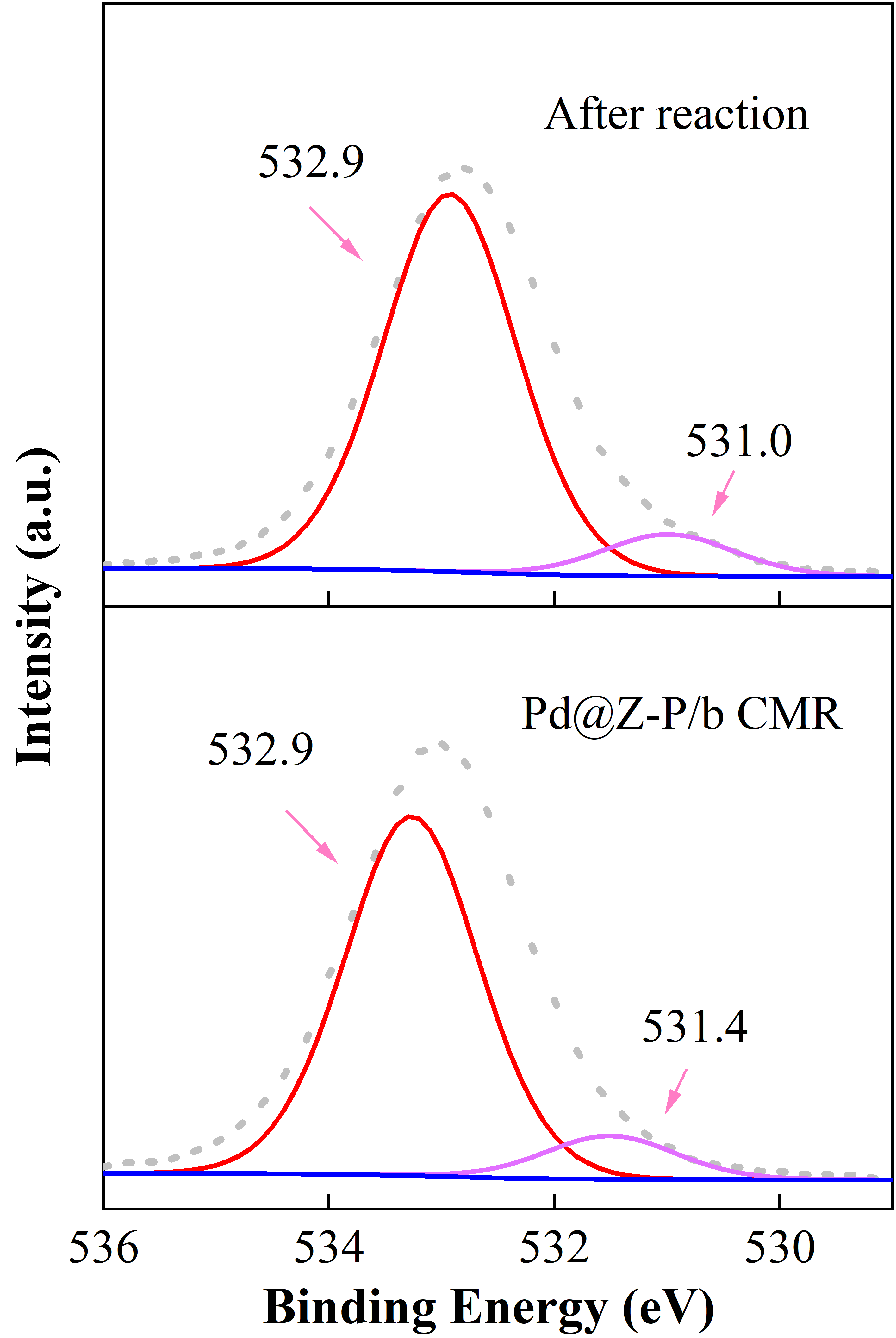


Figure S17. High resolution XPS spectrum for O 1s of Pd@Z-P/b CMR

before and after 10 d operation.


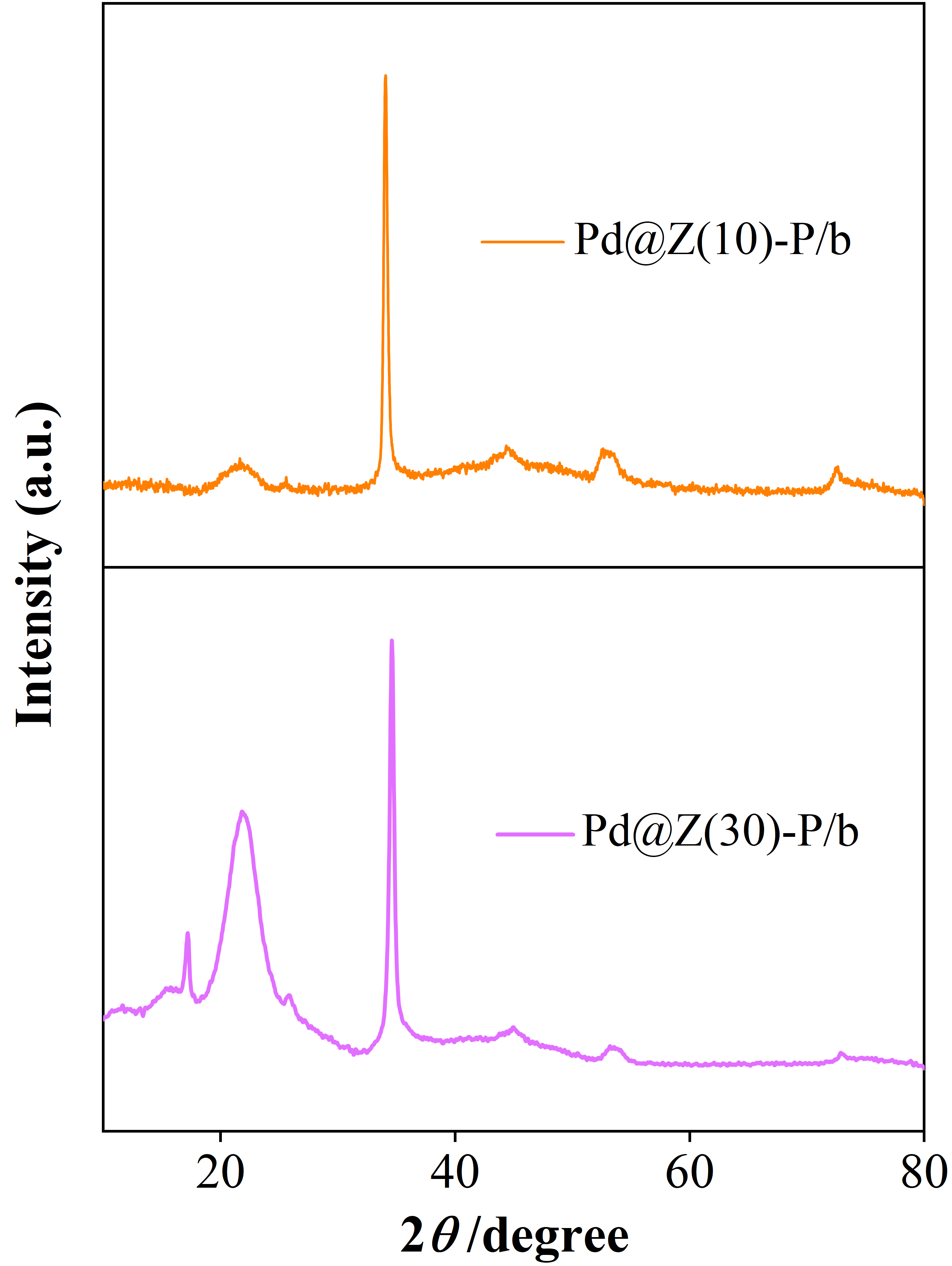


Figure S18. The XRD patterns for Pd@Z(10)-P/b CMR and Pd@Z(30)-P/b CMR samples.


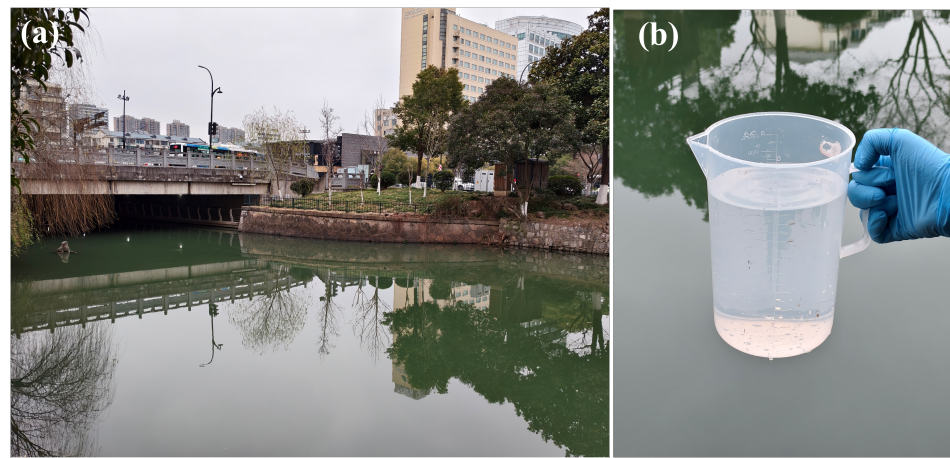


Figure S19. Location and photo of the Fengjia River water samples. The river water is highly turbid and contains a significant amount of suspended sediment.


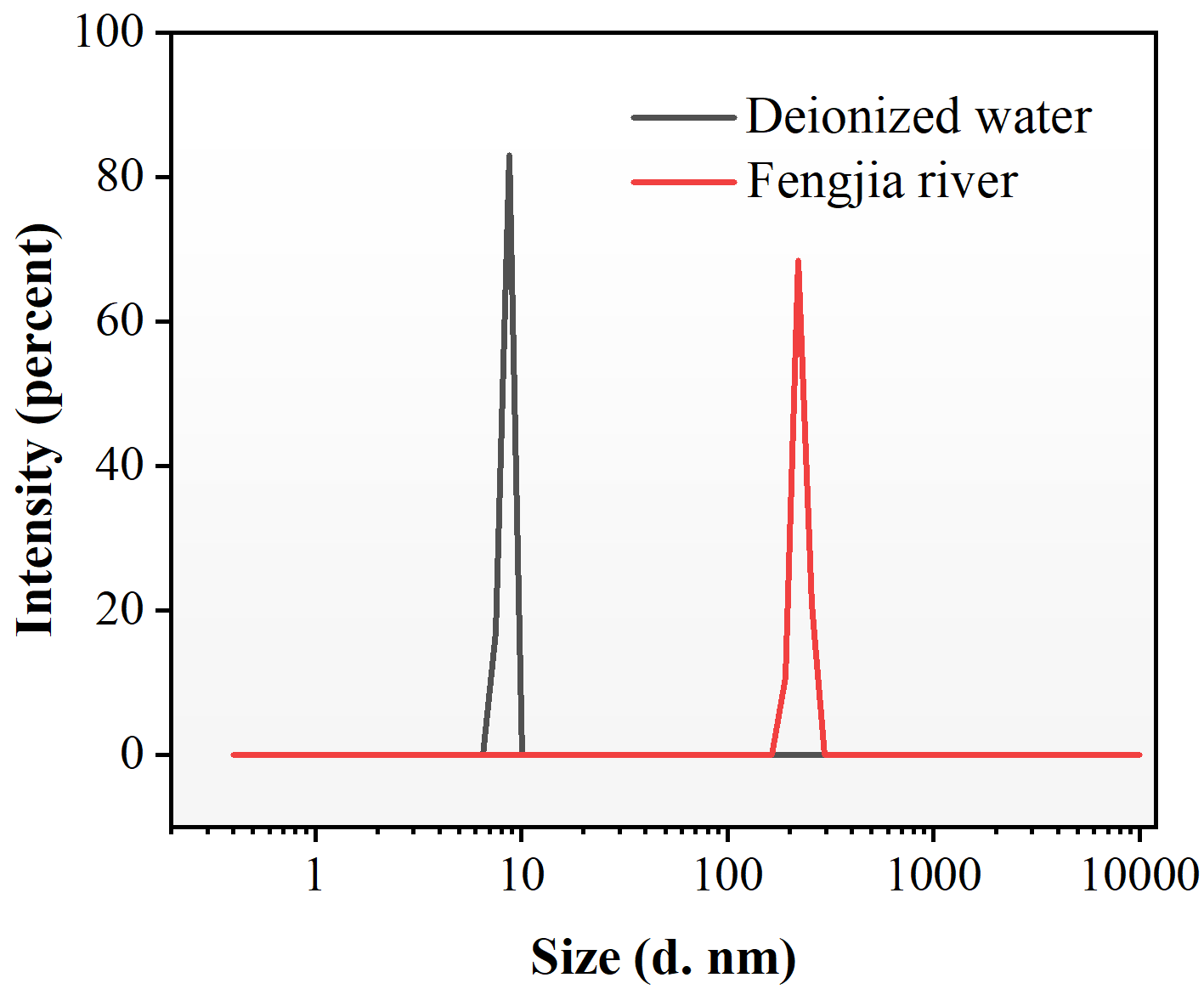


Figure S20. Comparison of the particle size distribution between the real river water and deionized water.

**Table S1.** ICP-MS results for the Pd@Z-P/b CMR and Pd-Z-P/b CMR.

| **Sample** | **Pd (wt%)** | **Co (wt%)** |
| --- | --- | --- |
| **Pd@Z-P/b CMR** | 0.0014 | 0.0663 |
| **Pd-Z-P/b CMR** | 0.0035 | 0.1032 |

**Table S2.** EXAFS data fitting results of Pd@Z-P catalysts.

| **Sample** | **Path** | **CN^a^** | **R(Å)^b^** | **σ^2^ (Å^2^)^c^** | **ΔE_0_(eV)^d^** | **R factor** |
| --- | --- | --- | --- | --- | --- | --- |
| **Pd@Z-P** | Pd-N | 3.81±0.18 | 2.03±0.01 | 0.0031 | 1.5 | 0.0090 |
|  | Pd-Pd | 1.03±0.22 | 2.71±0.02 | 0.0068 | -5.1 |  |

**Table S3.** Zeta potential (mV) of the Pd@Z-P/b CMR.

| **Sample** | **1** | **2** | **3** | **Average** |
| --- | --- | --- | --- | --- |
| **Zeta Potential (mV)** | -23.2 | -34.7 | -39.5 | -33 |

**Table S4.** Comparison of the removal efficiency of Pd@Z-P/b CMR with various other systems reported in the literature.

| **Materials** | **Catalyst content**  **(wt%)** | **Pollutants** | **Pollutant concentration (m****g·L⁻¹)** | **Duration (h)** | **Removal efficiency (%)** | **TOF (h^-1^)** | **Ref.** |
| --- | --- | --- | --- | --- | --- | --- | --- |
| **Fe_3_O_4_@C NPs** | 34 | MB | 200 | 2 | — | — | 1^[1]^ |
| **Pd NP/wood** | 0.19 | MB | 40 | — | 99.8 | 121.2 | 2^[2]^ |
| **Pd SACs-aerogel wood** | 1.7 | MB | 10 | — | 98.2 | — | 3^[3]^ |
| **Fe SACs-aerogel wood** | 0.3 | MB | 30 | — | 98.7 | — | 3^[3]^ |
| **ZIF-67@SC sponge** | 26.3 | MB | 80 | — | 86.99 | — | 4^[4]^ |
| **Pd-PDA/bamboo** | 0.17 | MB | 200 | 12 | 99.9 | — | 5^[5]^ |
| **Ag@CeO_2_ NCs** | 28 | 4-NP | 18.08 | 0.36 | 98 | 138.7 | 6^[6]^ |
| **Ag@CeO_2_ NCs** | 28 | 2-NA | 26.24 | 0.36 | 95 | 109.8 | 6^[6]^ |
| **Pd/TiO_2_-scaffolds** | 0.11 | 4-NP | 2000 | — | — | 161.4 | 7^[7]^ |
| **SA Co-N/C** | 2.45 | NPX | 10 | — | ~100 | 289.2 | 8^[8]^ |
| **CuNi_0.05_@OC** | Cu: 56.6  Ni: 0.4 | 4-NP | ~1391 | — | ~100 | 335.1 | 9^[9]^ |
| **Pd@Z-P/b** | 0.0014 | MB | 1000 | 120 | 97.5 | 1237.2 | This work |
| **Pd@Z-P/b** | 0.0014 | 4-NA | 800 | 240 | 94.3 | 2213.4 | This work |

**Table S5.** Total carbon (TC) and IC contents of Fengjia River water samples.

| **Sample** | **1** | **2** | **3** | **Average** |
| --- | --- | --- | --- | --- |
| **IC (mg·L⁻¹)** | 11.84 | 11.94 | 11.89 | 11.89 |
| **TC (mg·L⁻¹)** | 23.07 | 22.99 | 22.79 | 22.95 |
| **TOC = TC - IC** | — | — | — | 11.06 |

**References**

[1] X. Zhang, M. He, J.-H. Liu, R. Liao, L. Zhao, J. Xie, R. Wang, S.-T. Yang, H. Wang, Y. Liu, Fe_3_O_4_@C nanoparticles as high-performance Fenton-like catalyst for dye decoloration, *Chin. Sci. Bull.* **2014**, *59*, 3406.

[2] F. Chen, A.S. Gong, M. Zhu, G. Chen, S.D. Lacey, F. Jiang, Y. Li, Y. Wang, J. Dai, Y. Yao, J. Song, B. Liu, K. Fu, S. Das, L. Hu, Mesoporous, Three-Dimensional Wood Membrane Decorated with Nanoparticles for Highly Efficient Water Treatment, *ACS Nano.* **2017,** *11,* 4275–4282.

[3] G. Liu, Z. He, Y. Bai, Y. Li, C. Wang, J. Hu, X. Li, Y. Luo, D. Chen, 3D spiderweb-like nanowire networks loading single-atom catalysts in capillary array as high-efficiency microreactor, *Chemical Engineering Journal* **2023**, *453*, 139700.

[4] D. Liu, P. Jiang, X. Xu, J. Wu, Y. Lu, X. Wang, X. Wang, W. Liu, MOFs decorated sugarcane catalytic filter for water purification, *Chemical Engineering Journal.* **2022,** *431,* 133992.

[5] S. Yao, D. Guo, S. Han, Z. Fu, S. Lyu, J. Li, Y. Lu, Polydopamine-assisted immobilization of metallic nanoparticles confined regionally in bamboo microchannels as continuous-flow microreactors for enhanced catalysis, *Chemical Engineering Journal* **2024**, 492, 152327.

[6] Y. Shi, X. Zhang, Y. Zhu, H. Tan, X. Chen, Z.-H. Lu, Core–shell structured nanocomposites Ag@CeO2 as catalysts for hydrogenation of 4-nitrophenol and 2-nitroaniline, *RSC Advances* **2016**, 6, 47966.

[7] T. Liu, Y. Sun, B. Jiang, W. Guo, W. Qin, Y. Xie, B. Zhao, L. Zhao, Z. Liang, L. Jiang, Pd Nanoparticles Decorated 3D Printed Hierarchically Porous TiO_2_ Scaffolds for Efficient Reduction of Highly Concentrated 4-Nitrophenol Solution, *ACS Appl. Mater. Interfaces* **2020**, 12, 28100.

[8] Y. Qi, J. Li, Y. Zhang, Q. Cao, Y. Si, Z. Wu, M. Akram, X. Xu, Novel lignin-based single atom catalysts as peroxymonosulfate activator for pollutants degradation: Role of single cobalt and electron transfer pathway, *Applied Catalysis B: Environment and Energy* **2021**, 286, 119910.

[9] H. Yuan, M. Hong, X. Huang, W. Qiu, F. Dong, Y. Zhou, Y. Chen, J. Gao, S. Yang, Graphene Chainmail Shelled Dilute Ni─Cu Alloy for Selective and Robust Aqueous Phase Catalytic Hydrogenation, *Advanced Science* **2024**, 11.
